# Crosstalk between dysregulated amino acid sensing and glucose and lipid metabolism in colorectal cancer

**DOI:** 10.3389/fonc.2025.1665056

**Published:** 2025-08-29

**Authors:** Danning Wang, Shaomin Zou, Junkang Ding, Chang Gao, Jianwei Wang, Zhe Tang

**Affiliations:** ^1^ Department of Surgery, The Fourth Affiliated Hospital of School of Medicine, International School of Medicine, International Institutes of Medicine, Zhejiang University, Yiwu, China; ^2^ Department of Surgery, The Second Affiliated Hospital, Zhejiang University School of Medicine, Hangzhou, China

**Keywords:** colorectal cancer, amino acid sensing, metabolic reprogramming, glucose metabolism, lipid metabolism, mTORC1 signaling

## Abstract

Cancer cells reprogram the metabolism of glucose, lipids, and proteins (amino acids) to meet their energy needs during tumor initiation and progression. Amino acid sensing pathways play rucial roles in the progression and spread of colorectal cancer (CRC), but the crosstalk between these pathways and glucose and lipid metabolism has not been systematically elucidated. We summarize the roles of key amino acids in CRC, the corresponding nutrient sensors, the associated dysregulated signaling pathways, and their subcellular localization. Furthermore, we highlight how disrupted amino acid sensing forms an integrated regulatory network that modulates glucose and lipid metabolism through multiple signaling cascades. These insights reveal both opportunities for clinical translation and unresolved challenges in the field. We believe that this comprehensive review will stimulate further research in this emerging area and draw significant attention from both the scientific community and broader audiences. This review aims to identify new diagnostic markers, therapeutic targets, and prognostic indicators by enhancing the understanding of nutrient metabolic pathway interactions.

## 1 Introduction

Colorectal cancer (CRC) ranks among the most prevalent gastrointestinal malignancies and represents a significant health burden in China. In recent decades, there has been a marked increase in both CRC incidence and mortality, largely attributable to lifestyle shifts, such as high-fat, low-fiber diets and reduced physical activity. While genetic and environmental factors contribute to CRC pathogenesis, dietary and nutritional influences have emerged as pivotal modulators of disease risk, with a more pronounced effect in CRC than in many other cancers (1). Metabolic reprogramming is a hallmark of CRC malignancy, enabling tumor cells to fulfill increased energetic and biosynthetic demands essential for proliferation, metastasis, and therapy resistance (2). Key metabolic alterations include enhanced aerobic glycolysis, disrupted mitochondrial oxidative phosphorylation, upregulated fatty acid synthesis, and dysregulated amino acid and nucleotide metabolism. In particular, perturbations in glucose, lipid, and amino acid metabolism correlate with aggressive tumor phenotypes, such as unchecked growth and invasiveness (3). Amino acids act as critical regulators within metabolic signaling networks, influencing glucose metabolism, insulin secretion, proliferation, and differentiation (4). Cellular nutrient sensing—mediated by specialized receptors and intracellular pathways—detects fluctuations in amino acids, glucose, and lipids to maintain metabolic homeostasis (5, 6).

**Figure 1:**
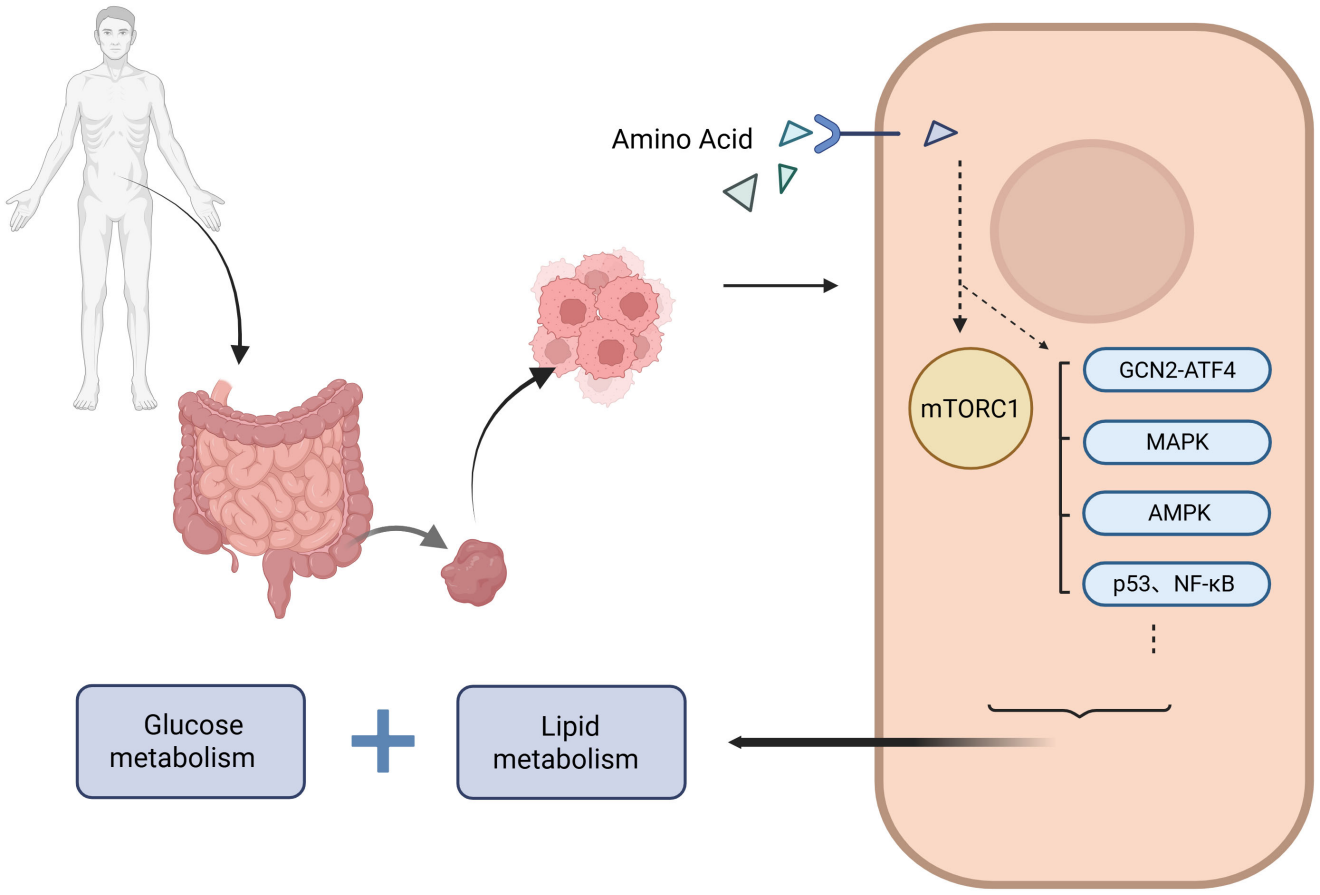
Dysregulated amino acid sensing pathways underpinning metabolic plasticity in colorectal cancer.

In the nutrient-poor tumor microenvironment, amino acid sensing pathways are often reprogrammed, facilitating tumor cell survival and growth under metabolic stress. This review summarizes the roles of key amino acids in CRC, the corresponding nutrient sensors, the associated dysregulated signaling pathways, and their subcellular localization. In CRC, aberrant amino acid sensing constitutes a well-defined regulatory network that directly governs glucose and lipid metabolism through distinct and intersecting signaling pathways. This network supports the metabolic adaptability of tumor cells and highlights potential molecular targets for therapeutic intervention. However, despite these insights, key mechanistic details remain unresolved, and their implications for clinical translation require further validation. This review consolidates recent advances in the field, aiming to facilitate targeted mechanistic investigations and encourage interdisciplinary research collaboration within the oncology and metabolic research communities. (as shown in **Figure 1**. Amino acid sensing pathways regulate metabolic reprogramming in CRC. Amino acids are transported into CRC cells and sensed by various intracellular pathways, including mTORC1, GCN2–ATF4, MAPK, AMPK, p53, and NF-κB. These pathways integrate nutrient signals and modulate metabolic reprogramming, affecting both glucose and lipid metabolism to support tumor growth and progression. Dysregulated amino acid sensing contributes to the metabolic plasticity of CRC cells, enabling adaptation to stress conditions such as nutrient deprivation and inflammation).

## 2 Amino acid metabolism in CRC

Dysregulated amino acid metabolism is a key feature of CRC, and promotes tumor proliferation and survival. CRC cells increase the expression of amino acid transporters to increase the uptake of essential substrates, fueling protein synthesis and metabolic signaling. Metabolomic profiling revealed altered pathways, including the tricarboxylic acid (TCA) cycle, the urea cycle, and glycolysis, and ornithine supported redox balance and biomass accumulation (7). Dietary factors significantly influence CRC development via amino acid metabolism. Patients often exhibit decreased circulating levels of glutamine, histidine, tryptophan, alanine, glycine, and citrulline, which may suppress tumor growth, whereas increased leucine, arginine, methionine, and aspartic acid levels are correlated with tumor progression (8–12). Aberrant metabolism of leucine, methionine, serine, and tryptophan enhances oxidative stress resistance, metabolic plasticity, and immune escape. CRC cells reprogram amino acid metabolism to meet bioenergetic and biosynthetic demands by catabolizing arginine, glutamine, and branched-chain amino acids through upregulated enzymes (13). These pathways represent potential therapeutic targets for dietary or enzymatic intervention.

### 2.1 Leucine

Leucine, one of the three branched-chain amino acids (BCAAs), plays a central role in regulating protein synthesis and intracellular signaling pathways. Epidemiological studies have demonstrated a positive association between high dietary leucine intake and increased mortality in CRC patients, particularly in males (14). BCAAs are transported into cells primarily via L-type amino acid transporters (LATs), and Solute Carrier Family 25 Member 44 (SLC25A44) specifically mediates their import into mitochondria. Once inside, BCAAs undergo catabolism, generating intermediates such as α-ketoglutarate (α-KG) and glutamate, which are essential for maintaining cellular energy homeostasis and supporting tumor cell proliferation (15). Beyond their metabolic functions, BCAAs also serve as nutrient-sensing molecules. Leucine activates mechanistic target of rapamycin complex 1 (mTORC1) through receptor-mediated pathways, thereby promoting anabolic processes and metabolic adaptation in cancer cells (16). In contrast, downregulation of mitochondrial Branched Chain Amino Acid Transaminase 2 (BCAT2) disrupts BCAA catabolism, resulting in the intracellular accumulation of BCAAs. This accumulation leads to sustained mTORC1 activation, which further accelerates CRC progression (17).

### 2.2 Arginine

Arginine plays a role in protein synthesis and is a precursor for tumor-associated metabolites such as polyamines, proline, and nitric oxide (NO) (18). High dietary arginine intake is epidemiologically linked to elevated CRC risk. In CRC cells, L-arginine uptake is mediated by the upregulated transporters Solute Carrier Family 7 Member 1 (SLC7A1),also known as Cationic Amino Acid Transporter 1 (CAT-1) and Solute Carrier Family 6 Member 14 (SLC6A14). Intracellularly, nitric oxide synthase (NOS) converts L-arginine to L-citrulline and NO, which further upregulates CAT-1, forming a feedforward loop that sustains arginine import. The L-citrulline generated in this process is recycled to L-arginine by argininosuccinate synthase (ASS) and argininosuccinate lyase (ASL). The regenerated L-arginine is subsequently converted by arginase into L-ornithine, which serves as a substrate for polyamine biosynthesis. In CRC, the upregulation of NOS, polyamine synthesis, and ornithine decarboxylase (ODC) is common and has been directly implicated in tumor growth and progression (19). Furthermore, arginine metabolism contributes to CRC development by supporting the interconversion of proline and glutamate and by activating the mTORC1 signaling pathway (20). In addition to its metabolic role, arginine also regulates gene expression epigenetically. Protein arginine methyltransferase 1(PRMT1) enhances CRC proliferation by asymmetrically dimethylating the non-POU domain-containing octamer-binding protein (NONO) at R251, facilitating epidermal growth factor receptor (EGFR) signaling activation and tumor progression (21–23).

### 2.3 Glutamine

Glutamine, the most abundant amino acid in plasma, is essential for CRC cell proliferation. Clinically, low glutamine levels correlate with poor prognostic indicators—such as advanced age, tumor progression, hypoalbuminemia, elevated Carcinoembryonic Antigen (CEA), and C-reactive protein (CRP)—and predict shorter survival outcomes (9). Oncogenic mutations often reprogram glutamine metabolism to support tumor viability. In CRC, glutamine is metabolized via glutaminolysis and the asparagine synthetase (ASNS) – Gamma-Aminobutyric Acid (GABA) shunt, both of which modulate the AMPK–mTORC1 signaling axis depending on glutamine availability (24). Under deprivation conditions, cells upregulate Glucose Transporter 1/3 (GLUT1/3) and reduce alanine and lactate synthesis. Combining glutamine with 2-deoxy-D-glucose enhances cytotoxicity, and compared with leucine, glutamine more significantly depletes TCA intermediates (25). Phosphatidylinositol-4,5-bisphosphate 3-kinase catalytic subunit alpha (PIK3CA) mutations further increase glutamine dependence by upregulating Glutamic Pyruvic Transaminase 2 (GPT2), altering glutamine flux (26). Additionally, mutations in Wnt/β-catenin, which are prevalent in CRC, reshape lipid and amino acid metabolism. The glutamine–α-ketoglutarate axis sustains Wnt signaling and differentiation, and under glutamine scarcity, cells consume more α-ketoglutarate to maintain this pathway, promoting tumor progression (27).

### 2.4 Methionine

One-carbon metabolism is critical for the proliferation and survival of both normal and cancerous cells due to its roles in DNA synthesis, repair, and methylation, which maintain genomic stability. Comparative analyses in CRC have identified methionine as a key metabolite associated with tumor progression (28).

As a dietary essential amino acid, methionine is converted into S-adenosylmethionine (SAM), the major cellular methyl donor involved in epigenetic regulation, polyamine synthesis, and mTORC1 signaling, thereby supporting macromolecule biosynthesis in tumor cells (29). Methionine restriction disrupts one-carbon metabolic flux, and when combined with 5-fluorouracil, it impairs nucleotide production and redox balance, suppressing CRC cell growth (30). This reflects cancer cells’ reliance on exogenous methionine, as they inefficiently regenerate it from homocysteine—a metabolic vulnerability termed methionine dependence or the Hoffman effect (31). This dependence is especially evident in tumor-initiating cells, which exhibit reduced endogenous synthesis and enhanced methionine uptake (32).

### 2.5 Other amino acids

#### 2.5.1 Aspartate–asparagine axis

In tumor cells, aspartate is generated through a transamination reaction between oxaloacetate and glutamate, which is catalyzed by aspartate aminotransferase. This reaction integrates amino acid degradation with nucleotide biosynthesis and cell proliferation, all of which are fundamental to cancer cell growth (33). Asparagine, synthesized from aspartate, plays two critical roles: it contributes to the biosynthesis of proteins and nucleotides, and it acts as an amino acid exchanger. Specifically, asparagine facilitates the import of extracellular serine, arginine, and histidine in exchange for intracellular asparagine, thereby activating the mTORC1 pathway and promoting protein synthesis (34). Therapeutically, blocking asparagine production with electron transport chain (ETC) inhibitors such as metformin, or depleting it using asparaginase or dietary restriction, has been shown to significantly reduce tumor cell proliferation (35).

#### 2.5.2 Histidine

Histidine, an essential amino acid involved in protein synthesis, one-carbon metabolism, and histamine/carnosine production, is metabolically reprogrammed in CRC. Epidemiological studies associate low circulating histidine with increased CRC risk (8). Catabolism via histidine ammonia-lyase (HAL) generates urocanate and downstream metabolites like glutamate, linking histidine to folate metabolism and redox regulation. In CRC, histidine catabolism consumes tetrahydrofolate(THF), impacting nucleotide synthesis and antifolate drug sensitivity (36, 37). Concurrently, histidine uptake is elevated through upregulation of LAT1 and SLC6A14, while lysosomal exporters (e.g. SLC15A4) recycle histidine intracellularly, linking to mTORC1 activation (38). Although a dedicated histidine sensor is lacking, histidine deprivation activates the GCN2–ATF4 stress axis, which is often bypassed in CRC (39). Thus, histidine scarcity could feed back through GCN2/ATF4 to modulate CRC metabolism. In summary, CRC cells rewire amino acid sensing and signaling networks so that histidine preferentially support growth.

#### 2.5.3 Serine

Serine, although a nonessential amino acid, plays a central role in one-carbon metabolism and lipid biosynthesis. It contributes to the generation of SAM, thus connecting glycolytic metabolism to methylation-dependent epigenetic regulation. This biochemical link is central to the Warburg effect and the methionine dependence observed in tumors, also referred to as the Hoffman effect (31). In CRC cells, serine racemase (SRR) catalyzes the conversion of serine into pyruvate and ammonia, which maintains mitochondrial function, elevates baseline reactive oxygen species (ROS) levels, supports cell proliferation, and inhibits apoptosis (40). The tumor suppressor gene Alcohol Dehydrogenase 1C (ADH1C) is frequently downregulated in CRC. When ADH1C overexpressed, suppresses the expression of Phosphoglycerate Dehydrogenase (PHGDH) and Phosphoserine Aminotransferase 1 (PSAT1), two key enzymes in the serine biosynthetic pathway, leading to lower intracellular serine levels and reduced tumor growth (41).

#### 2.5.4 Tryptophan

Tryptophan, an essential amino acid, plays an important role in CRC by modulating immune responses and interacting with the gut microbiota. Tryptophan-derived metabolites help maintain intestinal barrier integrity, regulate inflammation, and support the growth of beneficial gut microbes (42). Tumor cells often overexpress indoleamine 2,3-dioxygenase (IDO), which degrades tryptophan via the kynurenine (KP) pathway, promoting immunosuppressive conditions that facilitate tumor progression (43). The deubiquitinase Ubiquitin-Specific Protease 14 (USP14) further enhances this pathway by stabilizing components involved in tryptophan degradation (44). A decrease in tryptophan levels is correlated with an increase in immunosuppressive KP pathway metabolites (45). Additionally, downregulation of the serotonin transporter (SERT) interferes with mTOR serotonylation, indirectly enhancing tryptophan uptake and accelerating its conversion into kynurenine, a metabolite known to support immune evasion and tumor survival (46). Overall, the tryptophan metabolic pathway actively contributes to the inhibition of CRC development (as shown in **Table 1**).

**Table 1:** Key amino acids, their sensors/regulators, functions, roles in CRC, and potential therapeutic targets.

Amino acid	Amino acid sensor/regulator	Main functions	Role in CRC	Therapeutic targets
Leucine	Sestrin2 SAR1B LARS1	Stimulates protein synthesis & mTORC1; nutrient sensing	BCAT overexpression enhances VEGF secretion and mTORC1 activation, promoting angiogenesis & invasion	BCATs, mTORC1
Arginine	CASTOR1/2 SLC38A9 TM4SF5	Precursor for polyamines, NO, proline; regulates gene expression	PRMT1/5-mediated epigenetic changes promote CRC; NO enhances proliferation & invasion	NOS, PRMT1/5, ODC, ASS/ASL
Methionine	SAMTOR PRMT1	One-carbon donor for DNA methylation and nucleotide synthesis	Methionine dependence (Hoffman effect); poor regeneration from homocysteine	SAM pathway, dietary methionine restriction
Glutamine	SLC1A5(ASCT2) LAT1/SLC7A5–SLC3A2 LAPTM4b	Energy & nitrogen source; supports TCA and redox balance	Depletion linked to poor prognosis; PIK3CA & Wnt mutations increase dependence	ASNS, GPT2, GLUTs, combination metabolic blockade
Asp–Asn Axis	ATF4	Nucleotide synthesis; amino acid exchanger	Blocking asparagine synthesis significantly inhibits tumor growth	ETC inhibitors, asparaginase, dietary restriction
Histidine	GCN2	Carbon donor for TCA; modulates drug response and immune regulation	Loss of LHPP increases malignancy; METTL9 modulates immune suppression	LHPP, METTL9 (dual metabolic & immune targets)
Serine	ATF4	One-carbon metabolism, lipid & nucleotide synthesis	ADH1C suppresses PHGDH/PSAT1 expression, inhibits CRC growth	SRR, PHGDH, ADH1C
Tryptophan	GCN2	Regulates immunity and gut microbiota	SERT downregulation enhances uptake & immune evasion; KP promotes CRC	IDO, USP14, SERT

## 3 Amino acid sensors and organelles related to amino acid sensing

### 3.1 Amino acid sensors

Amino acid homeostasis relies on integrated intra- and extracellular sensing systems that regulate systemic metabolism (6). Beyond physiological maintenance, these pathways critically contribute to CRC progression by detecting amino acid fluctuations and activating downstream signals (47).

Leucine Sensing: The Sestrin protein family, which is composed of three members—Sestrin1, Sestrin2, and Sestrin3—functions as a critical regulatory unit in amino acid–sensitive signaling pathways (48). The key leucine sensors include Sestrin2, Secretion-Associated Ras-Related GTPase 1B (SAR1B), and Leucyl-tRNA Synthetase 1 (LARS1). Sestrin2 inhibits mTORC1 by binding GAP Activity TOward Rags 2 (GATOR2) under leucine-deprived conditions. Leucine binding induces its dissociation, enabling mTORC1 activation (49). Deprivation of amino acids such as isoleucine, lysine, glutamine, arginine, or cysteine induces Sestrin2 expression and suppresses mTORC1 (50). Similarly, SAR1B, a small GTPase involved in vesicular trafficking, operates as a leucine-sensitive regulator of mTORC1. It interacts with GATOR2 at a functional site analogous to that of Sestrin2. SAR1B similarly interacts with GATOR2 and is released upon leucine binding, promoting downstream signaling (51). Beyond amino acid sensing, SAR1B contributes to lipid transport processes; mutations that impair SAR1B function disrupt chylomicron secretion and are associated with gastrointestinal manifestations (52). In CRC, SAR1B is frequently overexpressed, with elevated expression correlating positively with tumor progression and inversely with patient survival outcomes (53). LARS1 senses cytoplasmic leucine and activates mTORC1 via RagD (54). Glucose deprivation leads to O-GlcNAcylation of LARS1, reducing its activity (55). Additionally, the L-type Amino Acid Transporter 1 (LAT1)- SLC3A2/Lysosomal-Associated Protein Transmembrane 4 Beta (LAPTM4b) complex mediates lysosomal leucine import, facilitating mTORC1 activation via V-ATPase. This interaction highlights the tightly regulated coordination between amino acid transport at the plasma membrane and subsequent lysosomal signaling events (56).

Arginine Sensing: Arginine is sensed by Cellular Arginine Sensor for mTORC1 Subunit 1/2 (CASTOR1/2) in the cytoplasm and Solute Carrier Family 38 Member 9 (SLC38A9) in lysosomes. CASTOR1 suppresses mTORC1 via GATOR2 binding under arginine deficiency, whereas arginine sufficiency triggers dissociation and pathway activation (49, 57). SLC38A9 detects lysosomal arginine and promotes mTORC1 via Ragulator–LAMTOR–Rag. Transmembrane 4 L Six Family Member 5 (TM4SF5) enhances this process by displacing CASTOR1 and relocalizing the complex to the lysosome (58, 59). TM4SF5—a protein embedded in the lysosomal membrane—modulates the coordination between cytoplasmic and lysosomal sensing mechanisms. Under arginine-sufficient conditions, TM4SF5 binds to SLC38A9, displacing its interaction with CASTOR1. This shift facilitates the relocalization of the sensing apparatus from the plasma membrane toward the lysosome, thereby amplifying arginine-dependent mTORC1 activation (60).

Glutamine and Methionine Sensing: Alanine, Serine, Cysteine Transporter 2 (ASCT2/SLC1A5) exports glutamine, supporting LAT1 (SLC7A5/SLC3A2)-mediated leucine import for mTORC1 activation (61). The high-affinity L-glutamine transporter ASCT2 and the heterodimeric SLC7A5/SLC3A2 bidirectional transporter are essential for mTORC1 pathway activity. They regulate the simultaneous efflux of L-glutamine from the cell and the transport of L-leucine/excitatory amino acids (EAAs) into the cell (62). SAMTOR binds SAM to inhibit mTORC1 via GATOR1 (63). Upon methionine sufficiency, SAM releases SAMTOR, while PRMT1 binds GATOR1, further promoting mTORC1 activation and linking methionine metabolism to nutrient signaling (64).

### 3.2 Organelles related to amino acid sensing

In solid tumors such as CRC, amino acid sensing depends on a highly coordinated network of organelles, each of which contributes to the regulation of mTORC1 signaling in a spatially and functionally distinct manner. At the plasma membrane, specific amino acid transporters mediate extracellular amino acid uptake, initiating intracellular nutrient-sensing pathways. Within the cytoplasm, lysosomes serve as the primary platform for interpreting amino acid availability and activating mTORC1 through lysosome-associated signaling complexes. Mitochondria modulate mTORC1 activity by integrating metabolic status and bioenergetic cues, whereas the ER-Golgi apparatus supports the localization and trafficking of mTORC1-related components required for mTORC1 activation. These organelles collectively construct a spatially defined and functionally integrated nutrient-sensing network that connects amino acid metabolism with mTORC1 signaling. This system underpins the metabolic flexibility, sustained proliferation, and treatment resistance that characterize malignant tumor cells (65, 66).

#### 3.2.1 Plasma membrane

Amino acid transporters embedded in the plasma membrane serve as the initial mediators of extracellular amino acid detection and entry into the cell (67). These proteins exhibit substrate specificity, enabling the selective import of individual amino acids into the cytosol, which is a critical step in activating nutrient-sensing signaling pathways. One key example is the LAT1 transporter, a heterodimer composed of SLC7A5 and SLC3A2, which is often enriched at the membrane and tightly regulates intracellular amino acid concentrations. After cellular uptake, specific cytosolic sensors—including Sestrin2 for leucine, CASTOR1 for arginine, and SAMTOR for S-adenosylmethionine—monitor intracellular amino acid levels and convey this information to central regulatory complexes such as mTORC1 (62, 68). These sensors not only detect amino acid availability but also, in some cases, contribute to intracellular amino acid trafficking, thereby linking metabolic inputs directly to signal transduction mechanisms (69).

#### 3.2.2 Lysosomes

Lysosomes, which are traditionally recognized as acidic organelles that mediate autophagy, endocytosis, and phagocytosis for macromolecule degradation and recycling, also function as signaling platforms transmitting molecular cues to the cytoplasm and nucleus. These roles are predominantly governed by transcription factor EB (TFEB), which colocalizes with mTORC1 on the lysosomal membrane (70). Recent studies have revealed that lysosomes serve as critical hubs for amino acid sensing, where they integrate nutrient availability with cellular growth signals to regulate mTORC1 activity. This regulation is fundamental for controlling cellular metabolism, energy balance, and proliferation (71). The initiation of amino acid sensing occurs within the lysosomal lumen (72). This observation suggests that the lysosome–mTORC1 axis may represent a conserved ancestral mechanism for nutrient sensing. The efficient delivery of amino acids into the lysosome is required for the rapid activation of mTORC1 and occurs via a vesicular transport pathway that does not rely on Akt signaling (73). Under nutrient-rich conditions, amino acids move bidirectionally between the lysosome and the cytosol. This dynamic exchange is regulated by specific membrane transporters and associated regulators (74).

Amino acid sensing begins within the lysosomal lumen, suggesting that the lysosome–mTORC1 axis represents an evolutionarily conserved mechanism for nutrient detection. The rapid activation of mTORC1 relies on the efficient import of amino acids into lysosomes via a vesicular transport pathway that operates independently of Akt signaling. Under nutrient-rich conditions, amino acids are dynamically exchanged between the lysosome and cytosol, a process mediated by specialized transporters and regulatory proteins. This bidirectional transport integrates signals from both cytosolic and lysosomal sources. Cytosolic sensors—such as Sestrin2 (leucine), CASTOR1 (arginine), and SAMTOR (S-adenosylmethionine)—cooperate with lysosomal components like the amino acid transporter SLC38A9 to relay nutrient status to mTORC1 (75). These signals converge on Rag GTPases, which are essential for recruiting and activating mTORC1 at the lysosomal membrane. The activity of this signaling network is tightly modulated by intracellular amino acid levels (72, 76). Rag GTPases serve as central regulators of amino acid-induced mTORC1 activation. The guanine nucleotide–bound state of mTORC1 controls its recruitment to the lysosomal surface. Although Rheb is not enriched in lysosomes under basal conditions, active Rag heterodimers transiently recruit mTORC1 to lysosomes, allowing Rheb-mediated activation (47, 77, 78). Following activation, mTORC1 disengages from lysosomes to phosphorylate downstream targets (79). Structural dimerization and conformational changes in Rag GTPases are essential for their activity and stability, thereby ensuring the precise regulation of mTORC1 signaling. Conversely, under amino acid deprivation, lysosomes act as transient storage compartments. Decreased intralysosomal amino acid content attenuates mTORC1 signaling, a process regulated in part by vacuolar-type H^+^-ATPase (V-ATPase), which maintains lysosomal pH and controls amino acid efflux (80). These regulators play key roles in promoting AMPK signaling through Axis Inhibitor (AXIN) (81). A distinct lysosome-associated sensing mechanism involves the transporter PQ Loop Repeat Containing 2 (PQLC2) and the Chromosome 9 Open Reading Frame 72 – Smith-Magenis Syndrome Chromosomal Region Candidate Gene 8 – WD Repeat Domain 41 (C9orf72–SMCR8–WDR41/CSW) protein complex, which is recruited to the lysosomal membrane during amino acid starvation. The membrane localization of this complex depends on the GTPase-activating protein (GAP) activity of Arf family proteins located on the Golgi and endosomal membranes, indicating functional communication between intracellular organelles during nutrient sensing (82).

#### 3.2.3 Mitochondria

Mitochondria are central to glucose and lipid metabolism, and regulate energy production, redox homeostasis, and apoptosis. Their function is maintained through quality control mechanisms, notably mitophagy, which removes damaged organelles (83). In cancer, mitochondrial dysfunction is common throughout tumorigenesis and is characterized by impaired TCA cycle enzymes, mtDNA mutations, and ETC defects. These changes increase ROS levels, disrupt redox balance, and, together with abnormal oncogene and tumor suppressor signaling, drive metabolic reprogramming that promotes tumor progression (84).

Mitochondrial metabolism is also regulated by nutrient-sensing pathways. mTORC1 promotes mitochondrial biogenesis and oxidative phosphorylation by activating transcriptional regulators such as Yin Yang 1 (YY1) and peroxisome proliferator-activated receptor gamma coactivator 1-alpha (PGC-1α) (85). Additionally, the mitochondrial pyruvate carrier (MPC) influences the activity of AMPK and mTORC1 by regulating the phosphorylation of their downstream targets. This modulation affects BCAA metabolism, linking mitochondrial pyruvate transport to broader nutrient-responsive metabolic control (86).

#### 3.2.4 The endoplasmic reticulum

The endoplasmic reticulum (ER) is a crucial organelle responsible for protein biosynthesis, including proper folding, intracellular trafficking, and turnover. While mTORC1 itself is not anchored to the ER membrane, several ER-linked pathways exert regulatory control over its activation. A portion of Rheb—a key upstream activator of mTORC1—associates transiently with the ER, where it facilitates mTORC1 activation through diffuse, nonspecific interactions with ER membranes (87). Upon stimulation by extracellular amino acids, the adaptor protein Fab1, YOTB, Vac1 and EEA1 (FYVE) and Coiled-Coil Domain Containing 1 (FYCO1) is targeted to lysosomal membranes, where it enhances physical contact between lysosomes and the ER. This process is mediated through protrudin, a PtdIns3P-binding ER (PtdIns3P) protein, and ultimately promotes the peripheral redistribution of lysosomes, a spatial rearrangement that supports mTORC1 signaling activation (88).

In parallel, the ER-anchored protein Aster-C (GRAMD1C) acts as a nutrient-sensitive suppressor of mTORC1. During amino acid deprivation, Aster-C binds and retains the mTORC1–GATOR2 complex on the rough ER, thereby preventing its activation. Upon nutrient reavailability, this interaction is disrupted, allowing the complex to be packaged into Coat Protein Complex I (COPI) -coated vesicles and delivered to the lysosomal membrane, where it re-engages with the mTORC1 activation machinery (89).

#### 3.2.5 Golgi apparatus

**Figure 2:**
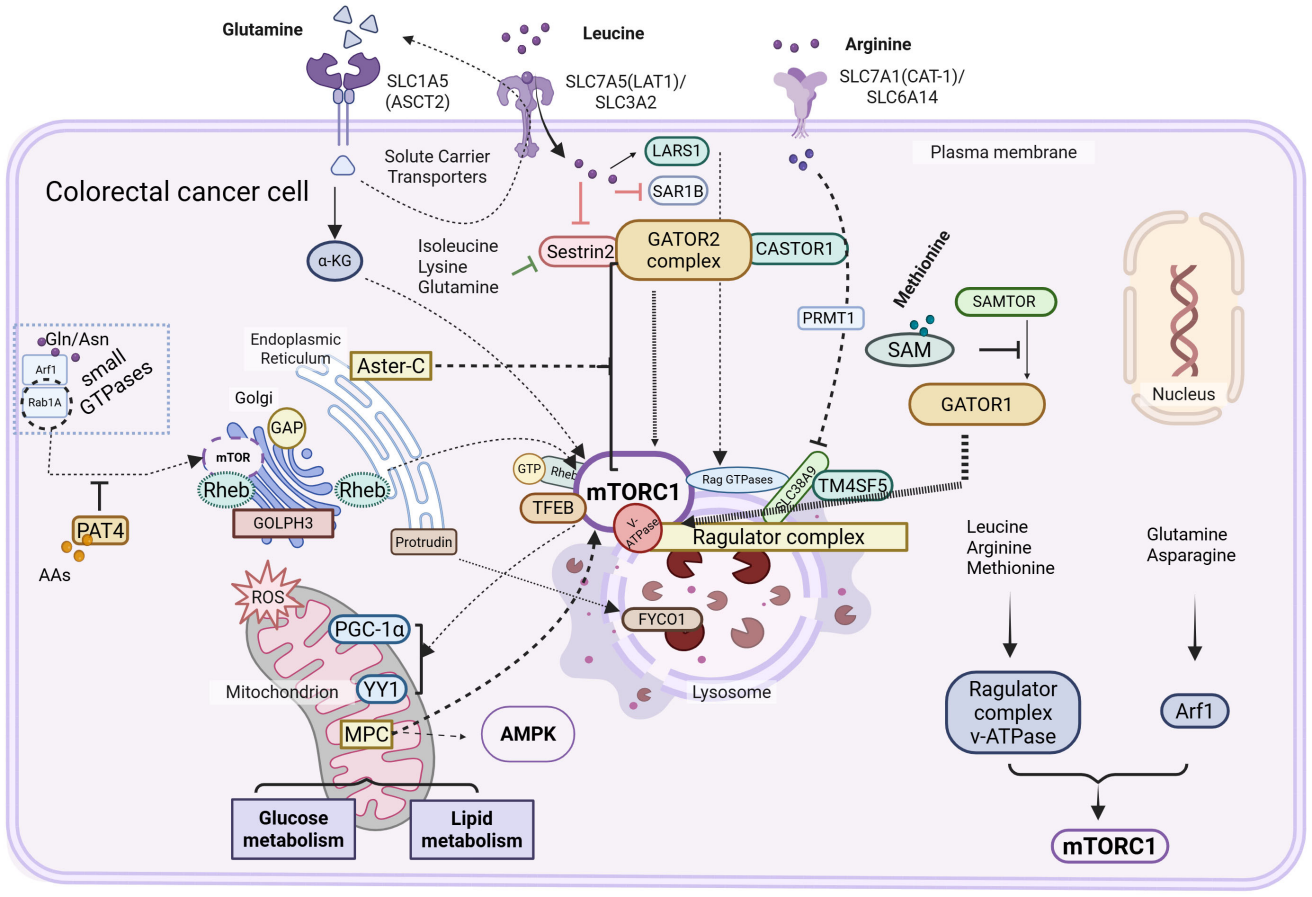
Amino acid sensing–mTORC1 signaling axis in the metabolic reprogramming of colorectal cancer cells.

Emerging evidence reveals that in the absence of lysosomal mTOR, an alternative mTOR pool localizes to the Golgi apparatus, indicating that both organelles independently mediate amino acid sensing. Unlike the canonical lysosome-dependent mechanism, Golgi-associated mTORC1 activation is regulated by small GTPases, notably ADP-ribosylation factor 1 (Arf1) and Rab1A (90). Golgi vesicular trafficking and structural integrity rely on guanine nucleotide exchange factors (GEFs), such as Golgi Brefeldin A Resistant Guanine Nucleotide Exchange Factor 1 (GBF1), which activate Arf GTPases essential for membrane trafficking (91, 92). While leucine, arginine, and methionine activate mTORC1 via a Rag GTPase–dependent pathway involving the Ragulator complex and v-ATPase, glutamine and asparagine act through a Rag-independent, Arf1-dependent mechanism (93–95). Rab1A, which is frequently overexpressed in CRC, promotes mTORC1 signaling by facilitating Rheb-mTORC1 association at the Golgi in response to amino acid stimulation (90). Similarly, Proton-Assisted Amino acid Transporter 4 (PAT4)—also upregulated in CRC—interacts with Rab1A and mTORC1, functioning as both an intracellular amino acid transporter and an alternative mTORC1 modulator (96). Additionally, Golgi Phosphoprotein 3 (GOLPH3) has been identified as a Golgi-resident protein involved in mTOR signaling regulation (97). (as shown in **Figure 2**. Amino acid sensing and mTORC1 signaling regulate metabolic reprogramming in CRC cells. This schematic illustrates how CRC cells sense and respond to amino acid availability through various solute carrier transporters (e.g., SLC1A5, SLC7A5, SLC7A1) and intracellular signaling pathways. Amino acids such as glutamine, leucine, arginine, and methionine activate the mTORC1 complex through a network of upstream regulators including GATOR1/2 complexes, CASTOR1, SAMTOR, Sestrin2, and LARS1. The Rag and Rheb GTPases, together with lysosomal components (e.g., TM4SF5, FYCO1, v-ATPase), contribute to the spatial and functional activation of mTORC1 at the lysosomal surface. Additionally, the Golgi-associated GOLPH3 and small GTPases (e.g., Arf1, Rab1A) modulate mTORC1 activity. Activated mTORC1 orchestrates transcriptional and metabolic responses by regulating downstream effectors such as TFEB and PGC-1α, which in turn affect glucose and lipid metabolism. Crosstalk with other signaling pathways, including AMPK and YY1, further integrates nutrient status to support CRC cell growth and survival).

CRC cells exhibit distinct metabolic dependencies on specific amino acids, reflecting a reprogrammed nutrient-sensing architecture centered on mTORC1 signaling. Leucine activates mTORC1 through cytosolic sensors such as Sestrin2, SAR1B, and LARS1, thereby promoting protein synthesis and sustained proliferation. Arginine sensing is mediated by CASTOR1/2 in the cytosol and SLC38A9 within lysosomes, with TM4SF5 amplifying arginine-dependent mTORC1 activation by facilitating lysosomal relocalization of the sensing complex, which contributes to tumor growth and immune evasion. Glutamine, a central metabolic substrate in CRC, regulates mTORC1 activity indirectly by fueling LAT1-mediated leucine uptake via the ASCT2–LAT1 antiporter axis, highlighting its role as a metabolic gatekeeper. Methionine sensing, via the SAMTOR–PRMT1 axis, couples intracellular SAM levels to mTORC1 activation, linking one-carbon metabolism to nutrient signaling and epigenetic regulation. Collectively, these amino acid–responsive circuits converge on mTORC1 to establish a spatially and functionally integrated signaling network involving the plasma membrane, lysosomes, mitochondria, and Golgi apparatus. This network not only sustains anabolic growth and redox balance but also underlies the metabolic plasticity and therapeutic resistance of CRC cells.

## 4 Regulation of amino acid sensing and related mechanisms

Amino acid sensing is a fundamental biological activity in normal cells and plays a crucial role in the dysregulation of these pathways during tumor development and progression (98). In cancer, the mTORC1 and AMPK pathways are the two primary intracellular systems for sensing metabolic status. AMPK is activated under conditions of low intracellular ATP, reflecting energy deficiency, whereas mTORC1 is activated in response to sufficient amino acid levels. In CRC, amino acid sensing-related pathways primarily include the mTORC, GCN2-ATF4, MAPK, AMPK, p53 and NF-κB pathways. These pathways jointly regulate tumor cell metabolism and proliferation by modulating downstream anabolic and catabolic processes, and represent potential therapeutic targets.

### 4.1 mTORC pathway

mTOR, a serine/threonine kinase belonging to the PI3K-related kinase family, assembles into two distinct functional complexes: mTORC1 and mTORC2, depending on its interacting partners.

#### 4.1.1 mTORC1 pathway

mTORC1 is the better-characterized of the two and integrates various upstream inputs (99). Growth factor stimulation, oxidative or genotoxic stress, and cellular energy depletion regulate mTORC1 via the tuberous sclerosis complex (TSC1/2), whereas amino acid sufficiency activates mTORC1 through a TSC-independent mechanism (77). Full activation of mTORC1 requires both Rheb, which directly stimulates its kinase activity, and the RagA–RagC heterodimer, which facilitates lysosomal translocation of mTORC1, positioning it to interact with Rheb (100). This localization process is tightly regulated by amino acid sensors such as Sestrin2 and CASTOR1, which specifically detect intracellular leucine and arginine, respectively, and modulate Rag GTPase activity accordingly.

Leucine and arginine serve as potent activators of mTORC1. This pathway primarily involves leucine, arginine, and methionine. Leucine and arginine, two essential amino acids, effectively stimulate mTORC1 activity. In contrast, glutamine and asparagine activate mTORC1 through alternative mechanisms (76). In particular, Sestrin2 binds to leucine; this binding relieves its inhibitory interaction with GATOR2, thereby permitting Rag activation and mTORC1 translocation (101). In contrast, amino acids such as glutamine and asparagine stimulate mTORC1 through an alternative mechanism that is independent of Rag GTPases but dependent on Arf1. The exact molecular details of this alternative pathway remain incompletely defined. In CRC, hyperactivation of mTORC1 is frequently observed and is often attributed to mutations or deletions in components of the GATOR1 complex, a negative regulator of Rag signaling (102). mTORC1 activity is not solely regulated by amino acids. Other physiological signals—including intracellular ATP levels, growth factor signaling, oxidative stress, and hypoxia—also modulate ATP activity. Posttranslational modification of Rheb, such as ubiquitination, enhances its interaction with mTORC1 at lysosomes, thereby integrating nutrient- and growth factor–derived signals (99, 103). Several core regulatory complexes govern Rag–mTORC1 signaling. These include the vacuolar H^+^-ATPase (v-ATPase), the Ragulator scaffold, the GATOR1/2 complexes, and the folliculin (FLCN) complex (104). Additional regulatory proteins—such as mitogen-activated protein kinase kinase kinase 3 (MAP4K3), the autophagy adapter protein- Sequestosome 1 (p62/SQSTM1), and G-protein–coupled receptor 137B (GPR137B)—also modulate this axis (68).

When intracellular amino acid levels are sufficient, amino acid transporters such as solute carrier (SLC) family members and vacuolar-type H^+^-ATPase (v-ATPase) become activated. These transporters initiate conformational changes that promote the activation of the Ragulator complex on the lysosomal membrane (105).

The vacuolar H^+^-ATPase (v-ATPase), localized on lysosomal membranes, consists of ATP-hydrolyzing V_1_ and proton-translocating V_0_ domains. These subunits reversibly assemble in response to intracellular amino acid levels, regulating v-ATPase activity and amino acid homeostasis (106). Under amino acid deprivation, V_1_ translocates to lysosomes and assembles with V_0_, enabling ATP-driven proton translocation into the lysosomal lumen, which acidifies the lysosome. This acidification is essential for proper amino acid sensing and activation of mTORC1 (72). AKT isoforms further modulate v-ATPase activity, supporting rapid responses to nutrient availability (107). Mechanistically, the V_0_ domain interacts with Ragulator, while V_1_ binds reversibly to the V_0_–Ragulator complex. Lamtor5, a Ragulator subunit, links the v-ATPase to Rag GTPase recruitment, thereby facilitating mTORC1 signaling. Collectively, v-ATPase plays dual roles: as a downstream effector that mediates lysosomal acidification and as an upstream nutrient-sensing hub that integrates amino acid signals to regulate mTORC1 activity. Mechanistically, the V_0_ domain interacts directly with Ragulator complex, while the V_1_ domain associates reversibly with the preassembled V_0_–Ragulator complex during mTORC1 activation (108). Within the Ragulator, the Lamtor5 subunit physically binds to a specific v-ATPase component, thereby facilitating the recruitment and activation of Rag GTPases and subsequent mTORC1 signaling (109). In summary, v-ATPase functions both as a downstream effector executing lysosomal acidification under mTORC1 control and as an upstream nutrient-sensing component that relays amino acid availability to the mTORC1 signaling pathway.

Ragulator, also known as lysosomal adaptor and mTOR activator/regulator (LAMTOR), is a five-subunit scaffold composed of LAMTOR1 (p18), LAMTOR2 (p14), LAMTOR3 (MP1), LAMTOR4 (C7orf59), and LAMTOR5 (HBXIP). Among these, LAMTOR4 and LAMTOR5 are essential components required for amino acid–dependent regulation of the mTORC1 pathway (110, 111). The Ragulator complex functions as a platform that recruits and stabilizes Rag GTPases on the lysosome. Together, the Ragulator–Rag complex mediates the translocation of mTORC1 to the lysosomal surface, where it interacts with Rheb GTPase for subsequent activation. The p18 subunit of Ragulator plays a key role in anchoring the complex to the lysosome and organizing this signaling process (112, 113).TFEB facilitates mTORC1 activation via dimeric Ragulator–Rag assemblies, while amino acid deprivation induces p27 recruitment to Ragulator, suppressing mTORC1 and enhancing autophagy (114, 115). Under leucine deficiency, calnexin (CANX) is butyrylated at K525, promoting the LAMP2–Ragulator interaction and inhibiting Rag-dependent mTORC1 activation (116). TNF Receptor-Associated Factor 4 (TRAF4) - mediated ubiquitination of LAMTOR1 at K151 enhances mTORC1 signaling and contributes to inflammation-associated colorectal tumorigenesis (117).

The GATOR complex is localized on the lysosomal membrane and acts as a critical upstream regulator of mTORC1. It comprises two opposing subcomplexes: GATOR1, which inhibits mTORC1 via Rag GTPase inactivation, and GATOR2, which promotes mTORC1 activation by antagonizing GATOR1. WD Repeat Domain 24 (Wdr24), a core component of GATOR2, is essential for amino acid–induced mTORC1 activation and mediates mTORC1-independent lysosomal functions (118). During amino acid deprivation, the GATOR complex contributes to maintaining mTORC1 at the lysosomal membrane, ensuring rapid reactivation upon nutrient restoration (119).

Interleukin enhancer-Binding Factor 3 (ILF3) anchors the GATOR complex to lysosomes, facilitating mTORC1 regulation (120). Von Willebrand Factor C and EGF Domains (VWCE) negatively regulates amino acid–dependent mTORC1 activity by recruiting GATOR1 through the KPTN–ITFG2–C12orf66–SZT2 (KICSTOR) complex (121). GATOR2 mediates amino acid sensing by interacting with sensors such as Sestrin2 and CASTOR1, thereby modulating GATOR1 (122). Leucine-bound Sestrin2 inhibits GATOR2 and activates GATOR1, suppressing mTORC1 signaling under amino acid deprivation (101, 123). Different yet functionally coordinated pathways respond to various sources of amino acids by controlling mTORC1 activity on cellular organelles (124). Concurrently, the PI3K–AKT–mTORC1 axis integrates growth factor signaling with nutrient cues (125). Upon Receptor Tyrosine Kinase (RTK) activation, PI3K converts PIP2 to PIP3, which is counteracted by Phosphatase and Tensin Homolog (PTEN) (126). PIP3 recruits AKT, which phosphorylates Tuberous Sclerosis Complex 2 (TSC2), leading to Rheb activation and subsequent mTORC1 stimulation. Additionally, amino acids regulate the TSC2–Rheb axis via Ca²^+^/calmodulin binding to the TSC2 GAP domain, promoting protein synthesis (78). WHI2 serves as a negative regulator under amino acid starvation, inhibiting mTORC1 by inactivating RAG-like GTPases and modulating Protein Serine/Threonine Phosphatase 1 and 2 (Psr1/2), further linking amino acid availability to TORC1 suppression (127–129).

#### 4.1.2 mTORC2 pathway

Although mTORC2 remains less well- characterized than mTORC1 is, evidence indicates that it participates in nutrient sensing, particularly by linking amino acid availability to glucose metabolism (130). In contrast to mTORC1, which directly responds to nutrient levels, mTORC2 is primarily activated by growth factor signals via the PI3K pathway (131). However, amino acids can indirectly influence mTORC2 activity through upstream PI3K–Akt signaling (132). Under nutrient deprivation, cells may selectively activate either mTORC1 or mTORC2, depending on the specific type and severity of starvation (133).

Rictor, a core structural subunit of mTORC2, functions as a scaffold for substrate binding and is essential for amino acid sensing in T cells (134–136). LAT1 promotes mTORC2 activation by localizing to lysosomes and directly interacting with Rictor, thereby facilitating Akt phosphorylation (137). Similarly, cystine uptake mediated by SLC38A91 activates the p38–mTORC2 subunit mitogen-activated protein kinase-interacting protein 1 (Sin1)–mTORC2–Akt signaling cascade (138). During glutamine deprivation, Sestrin2 associates with mTORC2 while concurrently reducing mTORC1 activity. This observation suggests a compensatory relationship in which Sestrin2-mediated mTORC1 suppression may enhance mTORC2 activation under specific nutrient stress conditions (139). mTORC2, although less understood in this context, has been implicated in nutrient sensing, particularly in linking amino acid signals with glucose metabolism.

### 4.2 AMPK pathway

mTORC1 and AMPK are nutrient-sensitive kinases essential for maintaining metabolic homeostasis (140). AMPK modulates multiple downstream targets, including mTOR, Acetyl-CoA Carboxylase (ACC), Unc-51 Like Autophagy Activating Kinase 1 (ULK1), Mitochondrial Fission Factor (MFF), TSC2, and Regulatory Associated Protein of mTOR (RAPTOR), and inhibits mTORC1 by phosphorylating TSC2 and RAPTOR at the lysosomal membrane, where both pathways intersect (141–143). Under amino acid deprivation, AMPK attenuates mTORC1 activity to conserve energy while remaining responsive to amino acid fluctuations independent of mTORC1 (141, 144). AMPK also regulates NAD^+^ biosynthesis and is activated by upstream signals such as Liver Kinase B1 (LKB1), Insulin Receptor Substrate (IRS), and sesquiterpenoids; LKB1 is deacetylated by Sirtuin 1 (SIRT1), forming a metabolic feedback loop that supports tumor progression (101). AMPK activation is triggered by increased AMP/ATP or ADP/ATP ratios during energy stress and is primarily regulated by glucose but also senses amino acid levels (145). Calcium/Calmodulin-Dependent Protein Kinase Kinase Beta (CaMKKβ) mediates amino acid sensing, and the Cysteinyl-tRNA Synthetase (CARS)–CaMKK2–AMP-Activated Protein Kinase Gamma 2 Subunit (AMPKγ2) axis specifically detects cysteine deficiency (146, 147). Beyond these pathways, cells employ multilayered amino acid–sensing networks to integrate metabolic cues, modulating glycolysis and lipid metabolism.

### 4.3 GCN2–ATF4 pathway

The General Control Nonderepressible 2 (GCN2)–Activating Transcription Factor 4 (ATF4) signaling pathway is a fundamental mechanism for sensing amino acid deprivation and maintaining amino acid homeostasis. It is activated under conditions of intracellular or extracellular amino acid deficiency. GCN2, also known as eukaryotic initiation factor 2 alpha (eIF2α) kinase 4, functions as a stress-responsive kinase that is activated by the accumulation of uncharged transfer RNAs (tRNAs)—molecular indicators of amino acid insufficiency. This activation leads to GCN2 autophosphorylation and subsequent phosphorylation of eIF2α, which broadly suppresses cap-dependent protein translation while selectively enhancing the translation of specific transcripts, notably ATF4 (148, 149).

ATF4 orchestrates a transcriptional response that promotes the expression of genes involved in amino acid biosynthesis and transport. This regulation facilitates the replenishment of intracellular amino acid pools and supports cell survival during nutrient limitation. In cancer cells, ATF4-driven gene expression enhances the synthesis of nonessential amino acids and increases nutrient uptake, conferring an adaptive advantage in metabolically stressed microenvironments (69, 130).

GCN2 signaling also contributes to the suppression of mTORC1 activity. In CRC, glutamine deprivation triggers GCN2 activation, which reduces the transcription of the 47S ribosomal RNA (rRNA) precursor and inhibits mTORC1 signaling (150). Moreover, activated GCN2 phosphorylates F-box protein 22 (FBXO22), which mediates mTOR ubiquitination, thereby decreasing mTORC1 sensitivity to amino acid availability in both cell-based and animal models (151). Importantly, GCN2 does not directly sense essential amino acids, indicating that its activation is independent of essential amino acid depletion (152).

This pathway integrates with several nutrient stress-responsive modules. In the GCN2–ATF4–Sestrin2 axis, amino acid deprivation—including glutamine, arginine, methionine, and lysine—leads to AKT activation, with Sestrin2 expression induced specifically through ATF4-mediated transcription (50). In parallel, the GCN2–ATF4–Regulated in Development and DNA Damage Response 1 (REDD1) axis activates the AKT–mTORC2 signaling cascade under nutrient stress (153). Furthermore, GCN2 signaling converges with the GATOR2–GATOR1–KICSTOR–Rags complex, which regulates mTORC1 activity in response to leucine, arginine, and glutamine. When canonical amino acid sensing through Rag GTPases is impaired, GCN2-mediated activation of FBXO22 provides an alternative mechanism for mTORC1 inhibition (151).

### 4.4 MAPK pathway

The MAPK/ERK signaling cascade regulates a range of fundamental cellular processes, including proliferation, apoptosis, inflammation, angiogenesis, metastasis, and resistance to anticancer therapies. In CRC, this pathway is often dysregulated due to mutations, the overexpression of signaling components, or constitutive activation, all of which contribute to tumor progression (154). The MAPK signaling network consists of three main branches: extracellular signal-regulated kinase (ERK), c-Jun N-terminal kinase (JNK), and p38 MAPK. These kinases are activated in response to various intracellular and extracellular stressors, including nutrient fluctuations (155–157). In CRC, abnormal MAPK signaling promotes metabolic reprogramming, notably influencing glucose uptake, oxidative stress regulation, and fatty acid metabolism (158–160). Amino acid signals can initiate MAPK activation through multiple mechanisms. One mechanism involves Rag GTPases, which also play a critical role in mTORC1 activation. Another involves mTORC1-independent routes. The LAMTOR complex (late endosomal/lysosomal adaptor, MAPK and mTOR activator) facilitates these processes by recruiting Rag GTPases to lysosomes and anchoring Mitogen-Activated Protein Kinase Kinase 1 (MEK1) and ERK1/2 to lysosomal membranes. This localization enables spatially and temporally coordinated activation of both the mTORC1 and MAPK pathways, ensuring efficient integration of nutrient and growth factor signals to regulate proliferation and metabolic adaptation (161). Furthermore, specific amino acids—such as leucine, glutamine, and serine—can directly stimulate MAPK signaling via distinct sensor proteins. This amino acid–mediated activation enhances glycolytic activity and anabolic processes, thereby promoting cancer cell viability and proliferation under nutrient-deficient conditions.

### 4.5 Wnt/β-catenin pathway

Amino acid sensing mechanisms intricately interact with the Wnt/β-catenin signaling pathway, collectively coordinating metabolic reprogramming and tumor progression in CRC. This bidirectional crosstalk enables tumor cells to adapt to fluctuating nutrient availability and sustain proliferative and stem-like phenotypes.

Several amino acids activate mTORC1 through their specific intracellular sensors, the mTORC1 activation subsequently inhibits glycogen synthase kinase-3β (GSK3β), a key negative regulator of β-catenin, thereby promoting β-catenin stabilization and its nuclear translocation. As a result, the transcription of Wnt target genes involved in proliferation and metabolism is enhanced (49). Some downstream targets in the Wnt pathway (such as c-Myc and Cyclin D1) are also supported by mTORC1-mediated metabolism, promoting cell cycle progression and stemness maintenance (162). Additionally, amino acid transporters such as LAT1 (SLC7A5) are frequently upregulated in CRC, which further sustains mTORC1 and Wnt/β-catenin activity. LAT1 expression has been shown to be directly or indirectly regulated by β-catenin, forming a positive feedback loop that reinforces anabolic metabolism and stemness (163, 164). Glutamine metabolism also plays a central role in this axis. Through glutaminase-mediated conversion into α-KG, glutamine supports mitochondrial function and epigenetic homeostasis. α-KG, in turn, can stabilize β-catenin by modulating its demethylase activity and redox status (27). The inhibition of glutaminolysis has been reported to suppress β-catenin nuclear localization, suggesting that metabolic flux directly influences Wnt signaling output (165). Conversely, Wnt/β-catenin signaling regulates amino acid metabolism by transcriptionally activating key amino acid transporters and metabolic regulators. MYC, a canonical Wnt target gene, promotes the expression of genes involved in amino acid uptake and utilization, including SLC1A5, SLC7A5, and glutaminase (GLS) (166). Collectively, these findings highlight a reciprocal regulatory circuit between amino acid sensing and Wnt/β-catenin signaling that sustains CRC progression. Therapeutic strategies targeting this axis may disrupt metabolic–signaling feedback loops and improve outcomes in Wnt-driven tumors.

### 4.6 NF-κB pathway

A decrease in amino acid availability directly suppresses mTORC1 activity, initiating a cellular stress response that activates multiple proinflammatory signaling pathways, with the NF-κB pathway being prominent. Specifically, mTORC1 inhibition induces the stress-activated protein kinase (SAPK)/JNK cascade, which, under nutrient-deficient conditions, acts synergistically to enhance NF-κB signaling (167).

Simultaneously, amino acid deprivation triggers the integrated stress response (ISR), a conserved cellular mechanism for adapting to metabolic stress. During this response, protein kinase R (PKR)-like endoplasmic reticulum kinase (PERK) functions as a primary sensor that phosphorylates eIF2α upon amino acid scarcity. This phosphorylation increases the transcription of stress-responsive genes, including those regulated by NF-κB (168, 169).

Additionally, the absence of sulfur-containing amino acids, such as methionine and cysteine, activates the GCN2 kinase. GCN2 also phosphorylates eIF2α, reinforcing ISR signaling and selectively enhancing the translation of ATF4 mRNA, a key regulator of inflammatory responses (170). In CRC, reduced ATF4-dependent expression of asparagine synthetase (ASNS) combined with depletion of extracellular asparagine impairs tumor proliferation (171). Furthermore, amino acid deprivation disturbs metabolic homeostasis, leading to increased oxidative stress and the production of ROS, which further amplify NF-κB activation (172).

### 4.7 p53 pathway

The tumor suppressor protein p53 directly regulates amino acid metabolism by controlling the expression of amino acid transporters, such as Solute Carrier Family 7 Member 11 (SLC7A11), and metabolic enzymes, including glutaminase 2 (GLS2). In CRC cells, SLC family proteins mediate the uptake and intracellular transport of amino acids. Among these proteins, SLC7A11 functions specifically as a cystine importer and glutamate exporter, thereby maintaining the intracellular redox balance and influencing susceptibility to ferroptosis, an iron-dependent form of regulated cell death characterized by lipid peroxidation.

Ginsenoside Rh3 (GRh3) induces ferroptotic cell death in CRC by promoting iron-mediated lipid peroxidation through the Signal Transducer and Activator of Transcription 3 (STAT3)/p53/Nuclear factor erythroid 2–related factor 2 (NRF2) signaling axis. GRh3 also modulates amino acid metabolism by regulating SLC7A11 expression, which results in increased intracellular iron accumulation and enhances its antitumor efficacy (173).

During deprivation of serine, glycine, or glutamine, the circular RNA circMYH9 is upregulated. This upregulation enhances serine and glycine metabolism, promotes the glutathione (GSH) cycle, and regulates the NAD^+^/NADH ratio to maintain cellular redox homeostasis. Amino acid starvation activates p53, leading to increased intracellular ROS and the stabilization of hypoxia-inducible factor 1-alpha (HIF-1α) (174).

Several genes that are transcriptionally regulated by p53, including TP53-induced glycolysis and apoptosis regulator (TIGAR) and GLS2, contribute to maintaining redox equilibrium (175). Specifically, GLS2 catalyzes the conversion of glutamine to glutamate, supporting GSH biosynthesis, lowering ROS levels, and sustaining energy production. The p53-dependent upregulation of GLS2 protects cells from oxidative DNA damage, while the loss of p53 or GLS2 expression disrupts redox balance and promotes cell death (176, 177).

**Figure 3:**
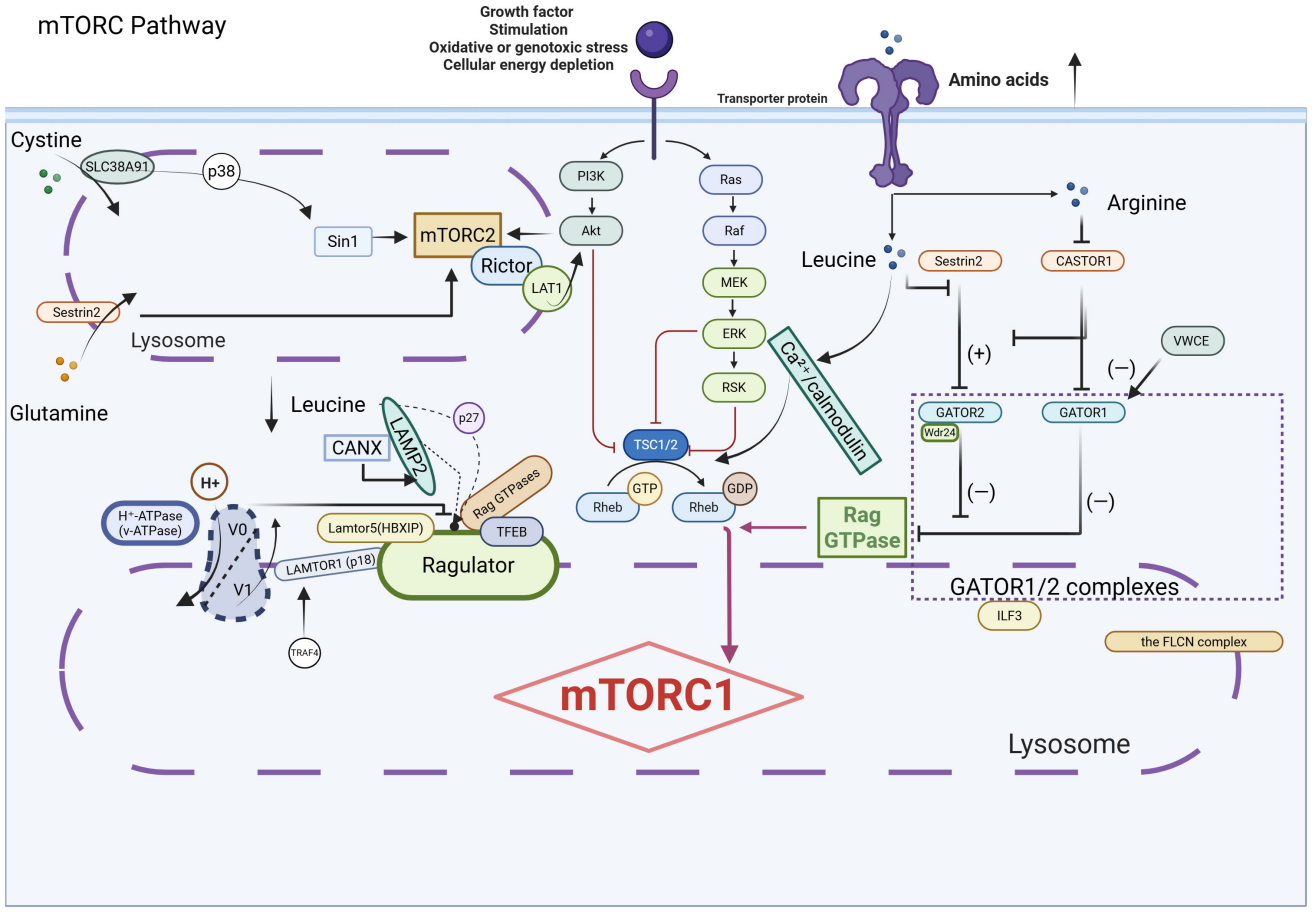
Coordinated regulation of mTOR signaling by amino acid sensing.

**Figure 4:**
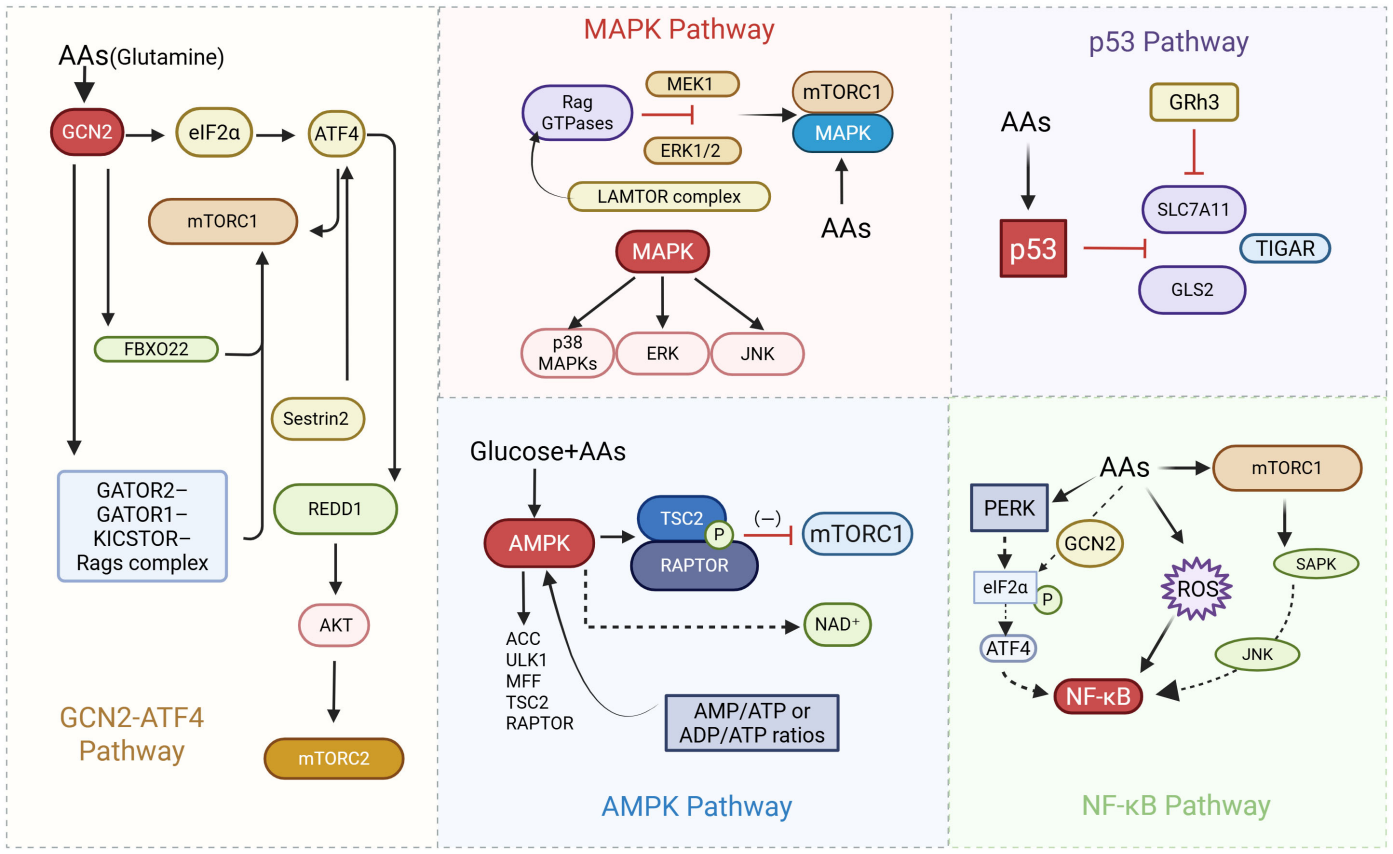
Mechanistic interplay of MAPK, p53, AMPK, and NF-κB pathways in nutrient and stress signaling.

Amino-acid sensing pathways such as mTORC1/2, AMPK, GCN2–ATF4, MAPK/ERK, Wnt/β-catenin, NF-κB, and p53 form a tightly interconnected network that governs CRC metabolism and growth. mTORC1 emerges as a central hub: it integrates oncogenic growth signals with amino acid inputs to drive anabolic programs. Clinical corollaries are evident – for example, CRCs with hyperactive mTORC1 often resist chemotherapy, yet acute amino acid (or dietary protein) restriction can blunt mTORC1 and re-sensitize tumors. This highlights mTORC1 (and its Rag-GTPase axis) as a therapeutic lynchpin. By contrast, mTORC2’s role in nutrient sensing remains less defined, though it interfaces with PI3K–AKT and has been implicated in metabolic control under stress. Other key pathways modulate or parallel mTORC1: energy stress activates AMPK (via LKB1/CaMKKβ), which phosphorylates ULK1 to induce autophagy and inhibits mTORC1. The GCN2–ATF4 arm senses amino-acid starvation (uncharged tRNAs) and triggers transcriptional programs to salvage nutrients; GCN2 also suppresses mTORC1 via mechanisms like FBXO22-mediated mTOR ubiquitination, providing an mTORC1-independent brake under amino acid deprivation. Parallel signaling cascades and transcriptional programs are deeply intertwined with metabolism. The MAPK/ERK pathway (often deregulated by KRAS/BRAF mutations in CRC) converges on metabolic regulators via scaffolds like the LAMTOR/Ragulator complex at lysosomes, coordinating mTOR and MAPK activation. Wnt/β-catenin (ubiquitously activated in CRC) likewise engages metabolic control: for example, β-catenin-driven c-Myc upregulates amino acid transporters (SLC1A5, SLC7A5/LAT1) and enzymes (GLS), linking Wnt activity to increased amino acid uptake and glutaminolysis. Conversely, leucine-activated mTORC1 can inhibit GSK3β, stabilizing β-catenin and fueling Wnt-dependent transcription – a reciprocal loop that reinforces proliferation. Notably, *in vitro* studies show Wnt activation raising intracellular arginine and histidine and inducing glycolysis (the Warburg effect), implying that Wnt signaling rewires amino-acid and glucose metabolism in tumors. Inflammation and stress pathways intersect as well: NF-κB is upregulated during amino acid shortage via JNK/SAPK and ISR crosstalk, promoting survival and cytokine production, while p53 regulates amino-acid handling through targets like SLC7A11 (cystine uptake) and GLS2 (glutamine catabolism) to maintain redox balance. For instance, p53-mediated induction of GLS2 enhances glutathione synthesis and protection from oxidative stress. This web of interactions underscores that multiple sensors jointly govern growth versus survival decisions in CRC cells.

(as shown in **Figure 3**. Regulatory network of the mTOR signaling pathway in response to amino acid and growth factor cues. This diagram illustrates the intricate regulation of mTORC1 by amino acids, growth factors, and intracellular signals. Amino acids such as leucine, glutamine, and arginine are sensed through transporter-mediated mechanisms and relay signals via the GATOR1/2 complexes, Rag GTPases, and the lysosomal v-ATPase–Regulator complex to activate mTORC1 at the lysosomal surface. Leucine inhibits Sestrin2, while arginine suppresses CASTOR1, both of which relieve repression of the GATOR2 complex, promoting mTORC1 activation. The FLCN complex and other lysosomal components (e.g., LAMTOR1/5, LAMP2) facilitate the recruitment and activation of mTORC1. Parallel to this, growth factor signaling through the PI3K–Akt and Ras–ERK pathways inactivates the TSC1/2 complex, promoting Rheb-mediated activation of mTORC1. Additionally, mTORC2, via Rictor and Sin1, modulates Akt and cytoskeletal dynamics. These coordinated pathways enable mTORC1 to integrate nutrient and mitogenic signals, orchestrating downstream anabolic processes essential for cancer cell proliferation and metabolism. as shown in **Figure 4**. Integration of Nutrient-Sensing and Stress-Response Pathways: Roles of MAPK, p53, AMPK, and NF-κB Signaling. Schematic representation of key signaling pathways involved in cellular responses to amino acids (AAs), glucose, and stress stimuli. The diagram highlights the interplay between other multiple pathways, including the MAPK pathway, p53 pathway, AMPK pathway, and NF-κB pathway. Key components such as GCN2, mTORC1, GATOR complexes, Rag GTPases, LAMTOR complex, and PERK are depicted, illustrating their roles in regulating cellular processes like translation, metabolism, and stress responses. Cross-talk between pathways (e.g., elF2a-ATF4, AMPK-TSC2, and ERK/JNK signaling) is emphasized, underscoring their integration in nutrient sensing and adaptive mechanisms).

## 5 Crosstalk between dysregulated amino acid sensing and glycolipid metabolism in CRC

Recent studies have increasingly emphasized that the macronutrient composition of the diet—specifically the levels of amino acids, glucose, and lipids—exerts a substantial influence on CRC onset and progression. These nutrients not only impact systemic metabolic homeostasis but also modulate the behavior of malignant cells by affecting their proliferation rates, invasive capacity, metastatic potential, and therapeutic responsiveness (178, 179). Within tumor cells, the metabolic pathways of amino acids, glucose, and lipids operate in a highly coordinated manner, and their interactions are dynamically regulated under both physiological and pathological conditions.

In particular, glucose and lipid metabolism are metabolically and functionally interconnected. Their biosynthetic and catabolic activities are jointly regulated according to the cellular energy requirements. AMPK serves as a central metabolic sensor that detects changes in the intracellular energy status and adjusts metabolic pathways accordingly (180). In parallel, mTORC1 integrates signals from amino acids, glucose, and lipids to orchestrate nutrient uptake and metabolic output. Dysregulation of mTORC1 signaling is a key contributor to both tumorigenesis and metabolic disorders (181). In CRC, tumor cells often depend on specific amino acids—such as glutamine and tyrosine—to support the biosynthetic and energy-generating processes required for sustained proliferation. Alterations in amino acid sensing or metabolism can lead to secondary reprogramming of glucose and lipid metabolic pathways. This interdependence highlights the integrated nature of nutrient metabolism in the oncogenic context and suggests that dysregulation of one pathway can drive compensatory changes in others.

### 5.1 Crosstalk between dysregulated amino acid sensing and glucose metabolism

A hallmark of tumor metabolic reprogramming is the preferential use of aerobic glycolysis—also known as the Warburg effect—in which cancer cells enhance glucose uptake and glycolytic throughput despite the presence of oxygen while concurrently downregulating mitochondrial oxidative phosphorylation. This adaptation supports redox homeostasis and generates intermediates essential for the nucleotide, amino acid, and lipid biosynthesis required during rapid proliferation.

In CRC, multiple oncogenic signaling pathways—including the PI3K/AKT, mTOR, MAPK, Wnt, and AMPK pathways—collectively modulate glycolytic activity. These pathways influence both the transcription and post-translational regulation of enzymes and transporters critical to glycolysis. Key transcriptional regulators, such as c-Myc, p53, and HIF-1, further reinforce this metabolic bias by upregulating genes that encode glucose transporters and glycolytic enzymes, thereby intensifying the glycolytic phenotype characteristic of CRC cells (182).

#### 5.1.1 mTORC pathway

mTORC1 functions as a master nutrient and energy sensor that integrates upstream signals to regulate glucose metabolism, cellular growth, and biosynthesis. In CRC, aberrant activation of mTORC1 enhances glucose uptake and accelerates glycolytic flux, thereby facilitating anabolic growth and tumor progression. Beyond its role in cancer metabolism, mTORC1 is essential for pancreatic β-cell viability and insulin production, acting through downstream mediators such as S6 kinase (S6K) and eukaryotic translation initiation factor 4E-binding protein 2 (4E-BP2) (181, 183). Rab1A, a small GTPase involved in ER-to-Golgi vesicle transport, potentiates leucine-induced mTORC1 activation by promoting lysosomal recruitment of the complex and stabilizing the transcription factor Pdx1. This links extracellular amino acid availability with insulin gene expression and β-cell function (184). GCN2 is activated under amino acid deprivation and suppresses mTORC1 activity by inducing Sestrin2 expression. Deletion of GCN2 results in Sestrin2 downregulation and TSC2-independent mTORC1 hyperactivation, which can be pharmacologically reversed by L-asparaginase (185). O-GlcNAcylation, a dynamic posttranslational modification controlled by O-GlcNAc transferase (OGT) and O-GlcNAcase (OGA), integrates nutrient cues—particularly glucose and amino acids—via the hexosamine biosynthesis pathway. In CRC, elevated O-GlcNAcylation of phosphoglycerate kinase 1 (PGK1) enhances its glycolytic activity and mitochondrial localization, thereby linking glycolytic flux to the TCA cycle. Notably, O-GlcNAcylation inhibits AMPK activity, whereas the AMPK-mediated phosphorylation of OGT establishes a feedback loop regulating nutrient sensing and energy homeostasis (186–188). Furthermore, mTORC1 can directly sense glycolytic intermediates such as dihydroxyacetone phosphate (DHAP), allowing its activation even under low-glucose conditions when DHAP is synthesized by triose kinase. The GATOR2–GATOR1–KICSTOR axis modulates this process, linking amino acid and glucose availability. As DHAP also serves as a precursor for lipid synthesis, this mechanism highlights the role of mTORC1 as a metabolic hub that bridges glucose and lipid metabolism (189). Complementarily, mTORC2 responds to metabolic stress by phosphorylating ribosomal S6K at Ser380 during amino acid or glucose deprivation, suggesting its involvement in maintaining cellular survival under nutrient-limited conditions (190). Leucine regulates mTORC1 through several mechanisms, including Sestrin2-mediated signaling, O-GlcNAcylation and phosphorylation of LARS1, and ubiquitin-dependent modulation of Sestrin2 by Ring Finger Protein 167 (RNF167) and STAM Binding Protein Like 1 (STAMBPL1). These interactions converge on the Rag GTPase–mTORC1 axis, ensuring fine-tuned control of mTORC1 activity and enhancing glycolytic output in CRC (55, 191). Similarly, arginine modulates mTORC1 through specific SLC transporters. SLC38A9, located on the lysosomal membrane, detects arginine and facilitates lysosomal leucine efflux. SLC25A29 transports arginine into mitochondria, promoting NO synthesis and driving glycolytic reprogramming and tumor aggressiveness (192–194).

#### 5.1.2 AMPK pathway

AMPK serves as a critical metabolic checkpoint that counteracts mTORC1 signaling during nutrient or energy deprivation. By promoting catabolic processes such as autophagy and inhibiting energy-intensive anabolic pathways, AMPK maintains the intracellular energy equilibrium. In CRC, AMPK is essential for metabolic recovery following amino acid starvation. Loss of AMPK impairs mTORC1 reactivation even when autophagy remains intact, underscoring its indispensable role in orchestrating post-stress adaptation (195, 196). Glucose deprivation further disrupts the AMPK-driven activation of the ULK1–Atg14–Vps34 complex, which is critical for autophagosome formation. This impairment compromises the ability of the cell to respond to mitochondrial dysfunction. Additionally, the LKB1–AMPK axis inhibits excessive ULK1 phosphorylation, thereby preserving energy homeostasis during mitochondrial stress. Under glucose-limiting conditions, AMPK upregulates the transcription factor Prospero homeobox 1 (PROX1), which stimulates BCAA catabolism. This process indirectly suppresses mTORC1 activity and curtails CRC progression, particularly in tumors with LKB1 deficiency (197). Although glycolysis is the primary energy-producing pathway in CRC, oxidative phosphorylation (OXPHOS) also contributes significantly to ATP generation. Mitochondrial damage leads to an increased AMP/ATP ratio, triggering AMPK activation. While this initially enhances glycolytic compensation, persistent mitochondrial dysfunction eventually results in metabolic collapse. Interestingly, the mitochondrial transporter SLC25A1 enhances OXPHOS by increasing oxygen consumption and promoting the activity of respiratory chain complexes I through V. This mechanism enables CRC cells to maintain energy production and viability under metabolic stress (198–200).

#### 5.1.3 Wnt/β-catenin pathway

The Wnt/β-catenin signaling axis, a central oncogenic pathway in CRC, plays a critical role in tumor metabolic remodeling. Within this pathway, GSK-3 acts as a metabolic effector, and its activity is modulated by upstream signals from insulin and Wnt ligands. Aberrant Wnt activation leads to β-catenin stabilization and nuclear accumulation, which subsequently drives the transcription of genes associated with enhanced proliferation, stemness, and therapy resistance. Clinically, high β-catenin expression is correlated with poor prognosis in CRC (201). KRAS mutations further potentiate Wnt-mediated metabolic alterations by upregulating the mitochondrial glutamine transporter Solute Carrier Family 25 Member 22 (SLC25A22). The resulting increase in glutamine import into the TCA cycle supports energy production and epigenetic remodeling. Specifically, the suppression of DNA demethylation by glutamine metabolism sustains Wnt target gene expression, thereby promoting stem-like traits and therapeutic resistance in tumor cells (202). In organoid models with APC deficiency, glutamine withdrawal exaggerates Wnt signaling, an effect that is reversible upon supplementation with α-KG, highlighting the regulatory influence of TCA intermediates on Wnt-driven transcription and differentiation (27). Under nutrient-restricted conditions, AXIN1, a scaffold protein within the β-catenin destruction complex, serves as a molecular interface between the Wnt and AMPK pathways. This crosstalk enables CRC cells to integrate energy-sensing mechanisms with oncogenic signaling, thereby fine-tuning their metabolic adaptation to fluctuating nutrient availability (189).

#### 5.1.4 NF-κB pathway

In CRC, amino acid deficiency leads to increased levels of intracellular ROS, which activate the NF-κB signaling cascade. Once activated, NF-κB transcriptionally upregulates key glucose metabolic genes, including GLUT1, hexokinase 2 (HK2), and lactate dehydrogenase A (LDHA), thereby enhancing glycolytic throughput and metabolic flexibility. Through direct transcriptional control, NF-κB promotes increased glucose uptake and sustains aerobic glycolysis in cancer cells (203–205). A distinct metabolic intermediary, α-hydroxybutyrate (α-HB)—a byproduct of BCAA catabolism—exerts tumor-promoting effects in CRC. α-HB facilitates the nuclear translocation of LDHA, which has been implicated in impaired insulin secretion. This metabolic interference may exacerbate CRC progression, particularly in patients with comorbid diabetes (206, 207). In parallel, NF-κB indirectly reinforces glycolytic reprogramming by inducing pro-inflammatory cytokines such as interleukin-6 (IL-6) and tumor necrosis factor-alpha (TNF-α). IL-6, in turn, activates Janus kinase (JAK), which phosphorylates and activates STAT3. The nuclear translocation of phosphorylated STAT3 enhances the transcription of glycolytic effectors including GLUT1, HK2, and LDHA (208, 209). Collectively, amino acid deprivation in CRC triggers ROS-mediated NF-κB activation, which not only reprograms glucose metabolism directly but also amplifies inflammatory signaling. This integrated metabolic-inflammatory axis facilitates the maintenance of glycolysis and supports tumor cell adaptation and survival under nutrient-limited conditions.

#### 5.1.5 p53

The tumor suppressor p53 orchestrates multiple metabolic pathways, including glycolysis, OXPHOS, and amino acid metabolism, through its role as a transcriptional regulator. In CRC, p53 enhances the expression of carboxymethylene butenolide homolog (CMBL), which destabilizes phosphofructokinase platelet-type (PFKP), a rate-limiting glycolytic enzyme. This suppression of glycolytic flux restrains tumor progression (210). Under serine-restricted conditions, p53 facilitates metabolic adaptation by modulating mTOR signaling and adjusting pyruvate kinase M2 (PKM2) levels. These regulatory changes reroute metabolic intermediates away from glycolysis toward the TCA cycle, preserving cellular survival during nutrient stress (211, 212). Loss of p53 function results in the upregulation of phosphoglycerate mutase 1 (PGAM1), which catalyzes the reversible conversion between 3-phosphoglycerate and 2-phosphoglycerate. This enzymatic shift enhances glycolytic throughput and contributes to redox homeostasis, especially under the hypoxic conditions common within the tumor microenvironment (213, 214).

#### 5.1.6 c-Myc

The oncogenic transcription factor c-Myc is a master regulator of metabolic reprogramming in CRC, modulating key pathways such as glycolysis, glutaminolysis, and nucleotide biosynthesis. Its expression is tightly governed by nutrient availability and oncogenic signaling (215). Under amino acid deprivation, the inhibition of mTORC1 facilitates the nuclear translocation of the GSK3–β-catenin complex, which suppresses c-Myc transcription. Conversely, under glutamine-deficient conditions, the serine synthesis pathway (SSP) is upregulated in CRC cells, thereby restoring c-Myc expression and promoting anabolic growth (216, 217). In addition, the calcium–calmodulin (Ca²^+^–CaM) complex enhances c-Myc activity by binding to its protein isoforms—such as c-Myc and N-Myc—predominantly in the cytoplasm. This interaction increases the transcriptional output of c-Myc and amplifies its oncogenic functions (218). Recent studies have also highlighted the regulatory role of long non-coding RNAs (lncRNAs) in c-Myc-driven metabolic adaptation. For example, LncRNA1764 has been shown to enhance the translation of the c-Myc protein, thereby promoting CRC cell survival, metastasis, and resistance to chemotherapeutic agents such as 5-fluorouracil by augmenting metabolic flux (219).

In CRC, the interplay between amino acid sensing and glucose metabolism is governed by a complex regulatory network. mTORC1 integrates nutrient signals to promote glycolysis and cellular growth, whereas AMPK counterbalances under metabolic stress by favoring catabolic processes and limiting mTORC1 activity. NF-κB further amplifies this glycolytic shift by linking amino acid deprivation and oxidative stress to inflammation-driven glucose metabolism.

### 5.2 Crosstalk between dysregulated amino acid sensing and lipid metabolism

Although increased glycolysis in tumor cells is well documented, recent research highlights the critical role of altered lipid metabolism in cancer progression, metastasis, and therapy resistance. Compared with normal cells, cancer cells exhibit distinct lipid metabolism, characterized by increased lipid uptake, elevated *de novo* lipogenesis, and enhanced fatty acid oxidation. These metabolic changes support rapid proliferation and are regulated by oncogenic signaling pathways that control lipid biosynthesis transcription factors (220). Additionally, tumor microenvironment factors—including hypoxia, upregulated fatty acid transporters, and interactions with stromal cells such as adipocytes and fibroblasts—augment exogenous fatty acid uptake, modifying the lipid profile of tumors (221).

In CRC, obesity is strongly associated with increased disease risk. Weight loss and improved insulin and leptin levels are correlated with a reduced incidence of CRC (222). CRC cells tightly regulate lipid uptake, synthesis, and degradation to maintain metabolic balance and support tumor growth under stress. Oncogenes such as KRAS, Myc, and APC transcriptionally regulate lipid metabolism, affecting tumor adaptation (223). Abnormal activation of lipid-processing enzymes by these oncogenes contributes to CRC initiation and malignancy. This reprogrammed lipid metabolism sustains energy production and membrane formation, while promoting tumor cell survival and invasiveness, thereby enhancing metabolic plasticity in CRC.

#### 5.2.1 mTORC1 pathway

In CRC, mTORC1 integrates amino acid availability with lipid metabolic reprogramming. Activated mTORC1 promotes lipogenesis by increasing the transcriptional activity of sterol regulatory element-binding protein 1 (SREBP1), which governs fatty acid and cholesterol synthesis. Lipin-1 phosphorylation facilitates SREBP1 proteolytic maturation and nuclear translocation. This process requires SREBP cleavage-activating protein (SCAP), which senses intracellular glucose and integrates its signals into lipid synthesis regulation (146). mTORC1 also couples glutamine metabolism with lipogenesis through SCAP-dependent coordination of glutaminolysis and glycolysis, maintaining anabolic flux during oncogenic stress. Activated SREBP1 induces the expression of fatty acid synthase (FASN) and ACC, which accelerate fatty acid and cholesterol synthesis and are linked to therapy resistance (224, 225). Glutamate serves as an allosteric regulator of ACC; thus, impaired glutamate sensing disrupts lipogenesis (226). Beyond the SREBP pathway, amino acid-induced mTORC1 activation involves the 4E-BP–peroxisome proliferator-activated receptor alpha (PPARα) axis, which regulates genes involved in fatty acid uptake, esterification, and β-oxidation. Transmembrane Protein 55B (TMEM55B) enhances mTORC1 by promoting lysosomal V-ATPase assembly, strengthening amino acid sensitivity (227). Branched-chain amino acids, particularly leucine, regulate mTORC1 via Sestrin2, influencing lipid oxidation and synthesis. Arginine sensing through CASTOR1 and SLC38A9 links amino acid and cholesterol signals to mTORC1 activation in CRC (228).

#### 5.2.2 AMPK pathway

Amino acid deficiency, particularly leucine depletion, is detected by nutrient sensors such as Sestrin2, which relay stress signals to activate AMPK. This activation is further triggered by an increased intracellular AMP/ATP ratio, reflecting disrupted cellular energy balance. AMPK functions as a central metabolic regulator that initiates adaptive responses to restore energy homeostasis (195, 229). Upon activation, AMPK inhibits lipid synthesis by phosphorylating SREBP1, a transcription factor essential for the expression of lipogenic genes. This phosphorylation prevents SREBP1 from entering the nucleus, resulting in decreased transcription of enzymes involved in fatty acid and cholesterol biosynthesis (230). Additionally, AMPK phosphorylates ACC at specific serine residues, leading to reduced enzymatic activity and lower production of malonyl-CoA, which is necessary for fatty acid synthesis (231, 232).

AMPK activation also promotes lipid breakdown by increasing the expression of genes involved in fatty acid oxidation. This effect is mediated through the elevation of intracellular NAD^+^ levels, which activates the NAD^+^-dependent deacetylase SIRT1. Activated SIRT1 stimulates PPARα, a key transcriptional regulator of mitochondrial β-oxidation and energy expenditure. Leucine influences this regulatory pathway by modulating adipokine secretion in adipose tissue and regulating the AMPK–SIRT1–PGC-1α axis, which controls mitochondrial biogenesis and lipid metabolism (233). Experimental studies have demonstrated that leucine supplementation increases SIRT1 expression via AMPK activation. This promotes the deacetylation and functional enhancement of PGC-1α, a transcriptional coactivator critical for oxidative metabolism (233). Moreover, the AMPK and mTORC1 pathways interact through direct and indirect phosphorylation mechanisms, allowing cells to coordinate metabolic adaptation and growth decisions in response to nutrient availability (234).

#### 5.2.3 GCN2–eIF2α–ATF4 pathway

In CRC, deprivation of key nutrients such as amino acids or glucose activates the GCN2 kinase. This activation leads to the phosphorylation of eIF2α, which enhances the selective translation of ATF4. Once induced, ATF4 coordinates transcriptional programs that regulate amino acid metabolism, cellular redox homeostasis, and the unfolded protein response (UPR), enabling tumor cells to cope with metabolic stress. Beyond its established roles in nutrient stress adaptation, ATF4 also modulates lipid metabolic pathways. It transcriptionally regulates key lipogenic enzymes, including fatty acid synthase (FASN) and stearoyl-CoA desaturase 1 (SCD1). Through this regulation, the GCN2–eIF2α–ATF4 signaling axis supports lipid synthesis and lipid droplet formation, contributing to cellular adaptation and survival under nutrient-limited conditions. Recent studies suggest that the ATF4-driven activation of lipogenesis is accompanied by a paradoxical suppression of lipid utilization, indicating a metabolic reprogramming strategy that favors biomass accumulation and tumor growth under stress (235). SCD1, a rate-limiting enzyme responsible for the conversion of saturated fatty acids (SFAs) to monounsaturated fatty acids (MUFAs), is primarily localized in the endoplasmic reticulum and plays a central role in maintaining membrane fluidity and lipid storage. Elevated SCD1 expression is closely associated with enhanced CRC cell proliferation and survival. However, during acute endoplasmic reticulum stress—a condition characterized by sustained eIF2α phosphorylation and elevated ATF4 expression—SCD1 transcription, along with that of other lipogenic genes, is significantly downregulated. This repression reduces glucose flux into lipids and reflects a temporary reallocation of metabolic resources away from lipid anabolism during stress adaptation (236). Among the lipid metabolism-related genes in CRC, FASN and SCD1 exhibited the most consistent and significant upregulation. The aberrant expression of these genes contributes to malignant progression and is correlated with increased metabolic activity and unfavorable clinical outcomes. SCD1 not only regulates the MUFA/SFA ratio but is also transcriptionally controlled by the liver X receptor (LXR), further implicating it as a key metabolic switch in CRC (160, 237). As a master enzyme of lipid desaturation, SCD1 influences not only fatty acid composition but also lipid droplet dynamics. It modulates lipolysis and autophagy-dependent lipid degradation, and its inhibition disrupts triglyceride accumulation, thereby impairing the lipid storage capacity essential for tumor sustenance (238). Pharmacological or genetic blockade of SCD1 has been shown to suppress lipogenesis and attenuate tumor progression, underscoring its therapeutic relevance. SREBPs, particularly SREBP1, are central regulators of genes involved in fatty acid and cholesterol biosynthesis. In CRC, the suppression of SREBP target genes is associated with reduced lipid production and impaired tumorigenicity (239). However, under nutrient-deprived or endoplasmic reticulum stress conditions, ATF4 overexpression can upregulate SREBP1, leading to increased triglyceride synthesis in a nutrient- and stress-dependent manner (240). Notably, the SREBP1–FASN–cholesterol biosynthetic axis has been identified as a critical mediator of radioresistance in CRC. Disruption of this pathway reduces cholesterol synthesis and enhances tumor cell sensitivity to radiotherapy, positioning it as a promising target for metabolic intervention (224).

#### 5.2.4 Wnt/β-catenin pathway

In CRC, disruption of amino acid sensing mechanisms can lead to aberrant activation of the Wnt/β-catenin signaling pathway. Under normal conditions, nutrient availability contributes to the regulation of Wnt activity; however, when nutrient-sensing is impaired, β-catenin accumulates abnormally in the cytoplasm and translocates into the nucleus. This nuclear localization initiates the transcription of oncogenic and metabolic targets, including c-Myc and Cyclin D1, thereby promoting tumor growth and metabolic reprogramming. Among the downstream targets of Wnt/β-catenin signaling, the BCAA transporter SLC7A5 is significantly upregulated in CRC. This upregulation reflects an increased reliance of tumor cells on amino acids such as leucine to sustain growth and biosynthetic demands (241, 242). Experimental evidence from CRC organoid models has demonstrated that β-catenin can directly bind to the promoter region of SLC7A5, enhancing its transcription and increasing intracellular BCAA concentrations. Elevated BCAA levels subsequently activate mTORC1, establishing a positive feedback loop—the Wnt–SLC7A5–mTORC1 axis—that integrates nutrient sensing with growth-promoting signaling (243). Functionally, SLC7A5 forms a heterodimeric antiporter complex with the glutamine transporter SLC3A2, enabling the bidirectional exchange of intracellular glutamine and extracellular leucine. This exchange mechanism is essential for leucine-dependent activation of mTORC1 (62). Once activated, mTORC1 phosphorylates and activates SREBP1, which in turn induces the transcription of key lipogenic enzymes such as FASN and ACC, thereby promoting *de novo* lipid synthesis (244). Moreover, Wnt/β-catenin signaling can further amplify mTORC1 activity through direct physical interaction with mTOR, synergistically enhancing Akt/mTOR pathway output and reinforcing the expression of lipogenic genes, including SREBP1, ACC, and FASN (245). This cooperation between oncogenic signaling and metabolic regulation underscores the capacity of CRC cells to adapt lipid metabolism in response to oncogenic and nutritional cues.

In addition to metabolic regulation, the Wnt/β-catenin pathway also drives tumor aggressiveness. Stimulation with Wnt family member 3A (Wnt3a), a canonical ligand of the Wnt pathway, increases CRC cell invasiveness and enhances metastatic potential. Notably, the overexpression of ATF4 has been shown to stabilize β-catenin, upregulate β-catenin and Cyclin D1 expression, and potentiate Wnt/β-catenin signaling, thus mimicking the pro-tumorigenic effects of Wnt3a (246).

#### 5.2.5 NF-κB pathway

Under conditions of amino acid deprivation or dysfunctional nutrient sensing, CRC cells initiate stress-adaptive responses that converge on the activation of the NF-κB signaling pathway. As a central transcriptional regulator, NF-κB controls a broad spectrum of metabolic genes that facilitate tumor cell adaptation to nutrient-deficient environments, a key requirement for sustained survival and progression. Among NF-κB-responsive metabolic targets, carboxylesterase 1 (CES1) functions as a critical lipase that promotes fatty acid oxidation (FAO). By facilitating lipid catabolism and preventing the accumulation of intracellular triacylglycerols, CES1 contributes to lipid homeostasis and supports CRC cell viability under metabolic stress (247, 248). NF-κB also plays a compensatory role in lipid remodeling during SCD1 deficiency, a condition that reduces cellular MUFA levels and increases ceramide synthesis—changes that compromise membrane integrity and may impair CRC cell survival (249). In high-glucose environments, the activation of carbohydrate response element-binding protein (ChREBP) enhances SCD1 expression, thereby increasing MUFA production and concurrently downregulating PTEN. These metabolic alterations facilitate CRC cell migration, invasion, and metastatic potential (250). Beyond its canonical transcriptional roles, NF-κB signaling is further modulated through the noncanonical NF-κB pathway, which is regulated in part by NF-κB-inducing kinase (NIK). NIK contributes to CRC pathogenesis by promoting epithelial regeneration in the colonic mucosa and modulating inflammatory signaling (251). Additionally, acyl-CoA synthetase long-chain family member 1 (ACSL1), a known transcriptional target of NF-κB, plays a pivotal role in inflammation-related lipid metabolism. In CRC and other malignancies, ACSL1 augments mitochondrial FAO via the AMPK–Carnitine Palmitoyltransferase 1C (CPT1C)–ATP axis, thereby supporting both tumor cell proliferation and metastatic dissemination (252, 253). At the downstream convergence point of several metabolic pathways, CPT1A serves as the rate-limiting enzyme of mitochondrial fatty acid β-oxidation. CPT1A is tightly regulated by oncogenic signaling networks, including NF-κB, positioning CPT1A as a critical metabolic hub in CRC development and progression.

A broader metabolic context reveals that disruptions in amino acid availability—caused by insufficient uptake or altered metabolism of amino acids such as glutamine, leucine, arginine, or serine—initiate a complex network of adaptive signaling pathways. These include GCN2–eIF2α–ATF4, NF-κB, and mTOR–S6K, which collectively orchestrate lipid metabolic remodeling. A central node of this response involves SREBPs, which regulate the transcription of lipid biosynthetic and transport genes. This signaling convergence drives the coordinated expression of lipid metabolic effectors, including SCD1, FASN, CPT1A, and Fatty Acid Binding Protein 4 (FABP4), integrating anabolic and catabolic lipid processes such as *de novo* synthesis, fatty acid β-oxidation, and intracellular trafficking. This metabolic flexibility enables CRC cells to thrive under microenvironmental constraints characterized by nutrient scarcity, hypoxia, and inflammatory cues.

#### 5.2.6 PI3K/Akt pathway

Several intermediates derived from glucose metabolism act as essential substrates for lipid biosynthesis, underscoring the indirect yet critical involvement of the PI3K/Akt signaling axis in regulating lipid metabolic pathways. Although traditionally associated with cell growth and survival, Akt also directly influences lipid metabolism by upregulating the transcription of enzymes required for *de novo* synthesis of fatty acids and cholesterol (254). This regulatory effect is primarily mediated through the activation of mTORC1. The PI3K/Akt pathway enhances lipid anabolism by promoting the expression and maturation of SREBPs, which are master transcriptional regulators of lipogenic genes (145). Akt facilitates the nuclear translocation of SREBPs via SREBP cleavage-activating protein (SCAP) (146) while preventing their degradation by inhibiting GSK3 (147, 178).

Under conditions of prolonged essential amino acid deprivation—such as the loss of arginine or leucine—cells are capable of reactivating mTORC1 through a mechanism dependent on PI3K/Akt, independent of classical autophagic or proteasomal recovery pathways (255). This observation suggests that PI3K/Akt signaling functions as a nutrient-responsive sensor that sustains metabolic homeostasis under stress. In CRC, members of the SLC25, the largest group of mitochondrial metabolite transporters, have emerged as crucial modulators of tumor metabolism and immune microenvironmental dynamics. Notably, SLC25A7 is significantly overexpressed in CRC and is correlated with sustained activation of the PI3K/Akt/mTOR signaling cascade. Similarly, SLC25A1—also known as the mitochondrial citrate/isocitrate carrier (CIC)—is upregulated in CRC and plays a direct role in lipid metabolic reprogramming. By exporting citrate from mitochondria into the cytoplasm, SLC25A1 increases the pool of cytosolic acetyl-CoA, the foundational substrate for *de novo* lipogenesis, thereby facilitating anabolic growth and enhancing tumor cell survival (200).

## 6 Conclusions and outlook

Dysregulated amino acid metabolism is a hallmark of CRC, supporting tumor growth, metabolic reprogramming, and immune evasion. CRC cells increase amino acid uptake and reroute pathways to maintain energy production, biosynthesis, and redox balance. Elevated levels of leucine, arginine, methionine, and aspartate are linked to tumor progression, while glutamine and histidine are often depleted. Each amino acid plays distinct roles. Amino acid sensing, mediated by cytosolic (Sestrin2, CASTOR1, SAMTOR) and lysosomal (SLC38A9) sensors, regulates mTORC1 signaling across organelles such as lysosomes, mitochondria, ER, and Golgi. mTORC1 integrates nutrient cues, especially leucine and arginine, and is activated by Rag GTPases and Rheb. Glutamine and asparagine activate mTORC1 via an Arf1-dependent pathway. In contrast, AMPK serves as an energy sensor, inhibiting mTORC1 under stress. Other nutrient-responsive pathways—including GCN2–ATF4, MAPK, p53, and NF-κB—further modulate amino acid metabolism, redox homeostasis, immune responses, and inflammation. These signaling pathways form a coordinated regulatory network that actively reprograms glucose and lipid metabolism in colorectal cancer, thereby linking amino acid availability to tumor progression, metabolic plasticity, and resistance to therapy. This network-driven flexibility enables colorectal cancer cells to survive and proliferate under conditions of nutrient deprivation, hypoxia, and inflammatory stress, highlighting actionable metabolic vulnerabilities that may serve as targets for therapeutic intervention.

Taken together, CRC exhibits a complex metabolic reprogramming landscape that extends beyond the classical Warburg effect to include dynamic alterations in amino acid and lipid metabolism, and indicates a tight coupling among glucose, amino acid, and lipid metabolism in CRC cells (256, 257). Furthermore, the interplay between metabolic enzymes and signaling pathways orchestrates CRC metabolic plasticity. Among these, amino acid sensing emerges as a central regulatory hub, linking nutrient availability to tumor cell fate decisions. Nutritional interventions have shown potential to inhibit tumor growth and reverse resistance, highlighting the crucial role of metabolic-nutritional crosstalk in CRC progression. The regulatory networks governing metabolic crosstalk in CRC are not yet fully delineated. Core nutrient-sensing pathways, including mTORC1, AMPK, and GCN2, have been identified; however, their interdependencies—particularly involving feedback regulation, subcellular compartmentalization, and integration with other signaling cascades such as Wnt and p53—remain inadequately defined. For example, Sestrin2 has been shown to modulate both mTORC1 and mTORC2 in response to amino acid deprivation, but the specific molecular mechanisms underlying this dual regulation are unclear. Under glutamine-deprived conditions, Sestrin2 enhances cell survival by inhibiting mTORC1 and stabilizing mTORC2, which in turn alters lipid metabolic pathways. Similarly, the lysosomal v-ATPase–LAMTOR complex regulates mTORC1 activation and facilitates MAPK pathway recruitment; however, the timing and localization of these regulatory events are not well characterized. The roles of ISR kinases such as PERK and GCN2 in CRC remain incompletely understood, especially in terms of their modulation of NF-κB signaling and autophagy during amino acid scarcity. Additionally, how p53 mutations—frequently observed in CRC—impact amino acid-sensing mechanisms, particularly through redox regulation involving SLC7A11 and GLS2, has not been thoroughly investigated.

Several fundamental knowledge gaps continue to impede progress. Much of the current understanding is derived from *in vitro* studies or non-colonic tissues, which fail to recapitulate the nutrient heterogeneity and environmental complexity of the CRC tumor microenvironment. Critical modulators—including cancer-associated fibroblasts, microbial communities in the gut, and dietary composition—are likely to influence amino acid availability and sensing, yet their roles remain underexplored in the CRC context. Furthermore, it is not yet established whether specific amino acid sensors directly contribute to metastasis or treatment resistance. The therapeutic efficacy of combining dietary interventions (e.g., protein-restricted diets) with targeted metabolic inhibitors is also largely untested. Regulatory complexes such as FLCN, KICSTOR, and WHI2 may be involved in CRC-specific amino acid sensing, but their functional contributions have not been systematically evaluated. Moreover, the minimal or threshold concentrations of individual amino acids required to activate key metabolic pathways *in vivo* remain poorly defined.

Although targeting amino acid sensing pathways has gained research momentum, effective clinical translation in CRC is still limited. Several molecular nodes in these pathways present pharmacologically actionable targets. mTOR inhibitors, including both rapalogs and ATP-competitive compounds, have been investigated in clinical trials; however, their efficacy is often compromised by compensatory reactivation of signaling pathways such as AKT. Amino acid transporters—particularly SLC7A5, which is transcriptionally activated by oncogenic factors like c-Myc and HIF-2α—are upregulated in CRC and represent promising targets. Specific LAT1/LAT2 inhibitors are currently in development. In preclinical studies, inhibition of the GCN2–ATF4 stress axis (e.g., with GCN2iB) sensitizes CRC cells to nutrient deprivation. AMPK activators such as metformin and phenformin, which indirectly suppress mTORC1 activity, are also under clinical evaluation. While Wnt and inflammatory signaling pathways remain difficult to inhibit directly, their metabolic consequences—such as NF-κB–mediated amino acid catabolism or Wnt-dependent nutrient transport—may yield novel therapeutic targets.

Nonetheless, multiple translational barriers persist. Many inhibitors targeting amino acid transport systems or biosynthetic enzymes exhibit limited efficacy due to tumor heterogeneity and compensatory metabolic rewiring. Only a small number of metabolic inhibitors—such as those against GLUT1 or IDO1—have shown consistent clinical benefit. Predictive biomarkers for selecting patients, monitoring treatment efficacy, or quantifying metabolic fluxes are currently inadequate. Conventional 2D *in vitro* models fail to mimic the spatial and metabolic heterogeneity of CRC tumors (258). Although advanced modalities such as mass spectrometry imaging and hyperpolarized MRI improve spatial resolution, standard metabolomics techniques lack sufficient spatiotemporal accuracy to capture dynamic metabolic changes *in vivo*.

To address these challenges, future investigations should adopt integrative, systems-level strategies to map how amino acid sensing interconnects with glucose and lipid metabolism, with an emphasis on subcellular signaling events. Advanced technologies—including single-cell metabolomics, spatially resolved proteomics, and imaging platforms such as Positron Emission Tomography (PET), Matrix-Assisted Laser Desorption/Ionization (MALDI), and hyperpolarized MRI—combined with AI-guided metabolic flux analysis, can enable real-time, spatially explicit characterization of CRC metabolism. These approaches are essential for identifying regulatory bottlenecks and context-specific vulnerabilities in tumor metabolic networks. For clinical translation, development of next-generation inhibitors targeting amino acid transporters and metabolic enzymes must be accompanied by precision diagnostics. Integrating metabolic and clinical data at scale will facilitate discovery of robust predictive biomarkers and support the design of rational combination therapies. Longitudinal studies assessing metabolic remodeling during disease progression and treatment will be critical for establishing mechanistic causality. Large-scale metabolomic profiling across CRC patient cohorts may further uncover diagnostic or prognostic indicators and clarify subtype-specific metabolic dependencies.

Ultimately, integrating metabolic insights into clinical decision-making could transform CRC management. Personalized dietary and metabolic interventions, guided by validated metabolic biomarkers and stratification systems, may offer practical applications in precision oncology. Approaches centered on tumor-specific metabolic phenotyping and targeted biomarker validation have the potential to significantly improve therapeutic outcomes in colorectal cancer.
